# Supplementary material for: Is what’s unexpected undesirable? Investigating the expectedness and desirability of gender-stereotypic behaviors in men and women
Source: PLoS One. 2026 Apr 1;21(4):e0343921. doi: 10.1371/journal.pone.0343921 (PMC13042864; doi:10.1371/journal.pone.0343921)
Supplement: Attachment — Submitted filename: RefereeReport_PlosOne_25June.pdf [file pone.0343921.s001.pdf]

Referee Report

Is what's unexpected undesirable? Investigating the expectedness and desirability of gender-stereotypic behaviors in men and women

June, 2025

I recommend a "minor revise and resubmit" for the paper.

1 Overview

The paper primarily tests Expectancy Violation Theory (EVT) in the context of gender stereotype violations. The authors contrast EVT with Role Congruity Theory and propose a Unified Theory of Gender Norm Violations in previous work. To empirically test the theory's assumptions, the authors conduct six studies. Beyond examining gender-stereotypic behaviors in typical men and women, they include two studies addressing the intersection of race and gender, and two studies examining the intersection of employment status (successfully working) and gender.

The authors contribute to the competing predictions on the relationship between gender-typicality and behavior desirability by providing survey-based evidence. Rather than relying on trait typicality measures, they directly assess expectedness and specific behaviors, allowing them to more concretely capture gendered traits and biases while reducing ambiguity that may arise when the same trait encompasses both positive and negative behaviors.

2 Comments

- The authors provide detailed information on the design and participant composition for each of the six studies in the texts. Since the studies are complementary and comparable, I suggest creating a summary table to present this more clearly and succinctly. For example, rows could include: "Question Asked", "Hypothesis tested", "Number and Share of Female Participants", "Number and Share of White Participants", "Total Number of Participants", and "Average Age of Participants", with columns for each of the six studies. This would provide a more straightforward overview of sample sizes and demographics, and allow the main text to focus on differences across studies. For instance, in Study 2a and 2b, the gender imbalance is larger than in the other studies.
- As acknowledged in the "Limitations" section, participants are primarily White and the samples are not nationally representative. While it is good to be transparent about this issue, I wonder whether and how this limitation might affect the results. One approach to address this would be to replicate the studies with nationally representative

samples; however, obtaining such samples is not always feasible. Alternatively, some adjustments or controls might be applied to account for sample composition. For example, they "fit linear mixed models" to test hypotheses 1 and 3, which includes "random intercepts for participant ID and specific behavior". My understanding is that they (1) use random effects for participant ID to account for repeated measures within each person as each participant rated multiple behaviors; and (2) use random effects for specific behavior to account for the differences in the average levels of expectedness or desirability across behaviors. It would be helpful if the authors could briefly discuss whether any adjustments for sample composition are feasible within this modeling framework, and or what types and directions of bias might arise.

- A minor point: in Figures 3, 7, and 11, it is difficult to distinguish results by gender when printed in black and white. I suggest using different symbols (e.g., circles for men and triangles for women) to improve clarity.
