# Supplementary material for: Is what’s unexpected undesirable? Investigating the expectedness and desirability of gender-stereotypic behaviors in men and women
Source: PLoS One. 2026 Apr 1;21(4):e0343921. doi: 10.1371/journal.pone.0343921 (PMC13042864; doi:10.1371/journal.pone.0343921)

PLOS ONE

Is what's unexpected undesirable? Investigating the expectedness and desirability of gender-stereotypic behaviors in men and women

--Manuscript Draft--

**Manuscript Number:**	PONE-D-25-13132
**Article Type:**	Research Article
**Full Title:**	Is what's unexpected undesirable? Investigating the expectedness and desirability of gender-stereotypic behaviors in men and women
**Short Title:**	Perceptions of expectedness and desirability of gender-stereotypic behaviors
**Corresponding Author:**	Hannah B Eareckson<br>New York University<br>UNITED STATES OF AMERICA
**Keywords:**	Gender stereotypes, gender bias, gender norms, racial stereotypes
**Abstract:**	When men and women engage in counter-stereotypic behaviors, they often face penalties for violating gender norms; however, there is evidence that men and women are not always penalized, and are sometimes even rewarded for engaging in counter-stereotypic behaviors. Existing theories of reactions to counter-stereotypic individuals make competing assumptions about the relationship between behavior gender-typicality and desirability: while theories of gender congruity assume that counter-stereotypic behaviors are inherently undesirable, expectancy violation theory assumes that desirability is largely independent of gender-typicality. In six studies, we investigate how gender-typicality affects the expectedness and desirability of twenty gender-stereotypic behaviors. Our results suggests that while behavior expectedness differs according to gender-typicality, judgments of behavior desirability are largely consistent for typical men and women (Studies 1a-1b), Black and Asian men and women (Studies 2a-2b), and successful working men and women (Studies 3a-3b). These results suggest that what is counter-stereotypic is not inherently undesirable and support the application of both expectancy violation theory and the unified theory of gender norm violations to predict reactions to gender norm violations
**Order of Authors:**	Hannah B Eareckson
	Madeline E Heilman, PhD
**Additional Information:**	
**Question**	**Response**
**Financial Disclosure**<br><br>Enter a financial disclosure statement that describes the sources of funding for the work included in this submission. Review the submission guidelines for detailed requirements. View published research articles from *PLOS ONE* for specific examples.<br><br>This statement is required for submission and **will appear in the published article** if the submission is accepted. Please make sure it is accurate.	The author(s) received no specific funding for this work.

Powered by Editorial Manager® and ProduXion Manager® from Aries Systems Corporation

**Funded studies**<br>Enter a statement with the following details:<br>• Initials of the authors who received each award<br>• Grant numbers awarded to each author<br>• The full name of each funder<br>• URL of each funder website<br>• Did the sponsors or funders play any role in the study design, data collection and analysis, decision to publish, or preparation of the manuscript?<br><br>Did you receive funding for this work?	
**Competing Interests**<br><br>Use the instructions below to enter a competing interest statement for this submission. On behalf of all authors, disclose any competing interests that could be perceived to bias this work—acknowledging all financial support and any other relevant financial or non-financial competing interests.<br><br>This statement is required for submission and **will appear in the published article** if the submission is accepted. Please make sure it is accurate and that any funding sources listed in your Funding Information later in the submission form are also declared in your Financial Disclosure statement.<br><br>View published research articles from *PLOS ONE* for specific examples.	The authors have declared that no competing interests exist.

**NO authors have competing interests**<br><br>Enter: *The authors have declared that no competing interests exist.*<br><br>**Authors with competing interests**<br><br>Enter competing interest details beginning with this statement:<br><br>*I have read the journal's policy and the authors of this manuscript have the following competing interests: [insert competing interests here]*<br><br>* typeset	
**Ethics Statement**<br><br>Enter an ethics statement for this submission. This statement is required if the study involved:<br><br>• Human participants<br>• Human specimens or tissue<br>• Vertebrate animals or cephalopods<br>• Vertebrate embryos or tissues<br>• Field research<br><br>Write "N/A" if the submission does not require an ethics statement.<br><br>General guidance is provided below. Consult the submission guidelines for detailed instructions. **Make sure that all information entered here is included in the Methods section of the manuscript.**	The NYU IRB board approved the study (IRB-FY2016-479). Written consent was obtained.

**Format for specific study types**<br><br>**Human Subject Research (involving human participants and/or tissue)**<br>• Give the name of the institutional review board or ethics committee that approved the study<br>• Include the approval number and/or a statement indicating approval of this research<br>• Indicate the form of consent obtained (written/oral) or the reason that consent was not obtained (e.g. the data were analyzed anonymously)<br><br>**Animal Research (involving vertebrate animals, embryos or tissues)**<br>• Provide the name of the Institutional Animal Care and Use Committee (IACUC) or other relevant ethics board that reviewed the study protocol, and indicate whether they approved this research or granted a formal waiver of ethical approval<br>• Include an approval number if one was obtained<br>• If the study involved *non-human primates*, add *additional details* about animal welfare and steps taken to ameliorate suffering<br>• If anesthesia, euthanasia, or any kind of animal sacrifice is part of the study, include briefly which substances and/or methods were applied<br><br>**Field Research**<br><br>Include the following details if this study involves the collection of plant, animal, or other materials from a natural setting:<br>• Field permit number<br>• Name of the institution or relevant body that granted permission	
**Data Availability**<br><br>Authors are required to make all data underlying the findings described fully available, without restriction, and from the time of publication. PLOS allows rare exceptions to address legal and ethical concerns. See the PLOS Data Policy and FAQ for detailed information.	Yes - all data are fully available without restriction

A Data Availability Statement describing where the data can be found is required at submission. Your answers to this question constitute the Data Availability Statement and **will be published in the article**, if accepted.<br><br>**Important:** Stating 'data available on request from the author' is not sufficient. If your data are only available upon request, select 'No' for the first question and explain your exceptional situation in the text box.<br><br>Do the authors confirm that all data underlying the findings described in their manuscript are fully available without restriction?	
**Describe where the data may be found in full sentences. If you are copying our sample text, replace any instances of XXX with the appropriate details.**<br><br>• If the data are **held or will be held in a public repository**, include URLs, accession numbers or DOIs. If this information will only be available after acceptance, indicate this by ticking the box below. For example: *All XXX files are available from the XXX database (accession number(s) XXX, XXX.)*.<br>• If the data are all contained **within the manuscript and/or Supporting Information files**, enter the following: *All relevant data are within the manuscript and its Supporting Information files.*<br>• If neither of these applies but you are able to provide **details of access elsewhere**, with or without limitations, please do so. For example:<br><br>*Data cannot be shared publicly because of [XXX]. Data are available from the XXX Institutional Data Access / Ethics Committee (contact via XXX) for researchers who meet the criteria for access to confidential data.*<br><br>*The data underlying the results presented in the study are available from (include the name of the third party*	The data are held in a public repository through OSF - https://osf.io/mpfsy/

and contact information or URL).<br>• This text is appropriate if the data are owned by a third party and authors do not have permission to share the data.<br><br>* typeset	
Additional data availability information:	

Manuscript Click here to access/download;Manuscript;renamed_268dd.pdf

Full Title: Is what's unexpected undesirable? Investigating the expectedness and desirability of gender-stereotypic behaviors in men and women

Short Title: Perceptions of expectedness and desirability of gender-stereotypic behaviors

Hannah B. Eareckson

OrcID: 0000-0002-4033-7711

Madeline E. Heilman

New York University

Author Note

Correspondence concerning this article should be addressed to Hannah B. Eareckson, Department of Social Psychology, New York University, 6 Washington Place, New York, NY, 10003. Email: hbe217@nyu.edu

Declarations

Ethical approval and consent to participate

All participants were over 18 and provided informed consent to participate in these studies.

Human and animal ethics

New York University's Institutional Review Board reviewed these studies and provided ethical oversight and approval for all studies involving human participants.

Consent for publication

Both authors consent to the publication of this manuscript.

Availability of supporting data

All data and analyses are available at https://osf.io/mpfsy/?view_only=89be74a03d9a4decacb03c5143b62a5d

Competing interests

The authors have no competing interests on this manuscript.

Funding

Funding was provided by the host university, New York University, for all studies in this manuscript.

Authors' contributions

The studies in the article were developed, administered, and analyzed by the lead author, Hannah Eareckson. The second and senior author, Madeline Heilman, provided guidance and final approval throughout the entire process of designing the studies, analyzing the data, and writing the manuscript.

Abstract

When men and women engage in counter-stereotypic behaviors, they often face penalties for violating gender norms; however, there is evidence that men and women are not always penalized, and are sometimes even rewarded for engaging in counter-stereotypic behaviors. Existing theories of reactions to counter-stereotypic individuals make competing assumptions about the relationship between behavior gender-typicality and desirability: while theories of gender congruity assume that counter-stereotypic behaviors are inherently undesirable, expectancy violation theory assumes that desirability is largely independent of gender-typicality. In six studies, we investigate how gender-typicality affects the expectedness and desirability of twenty gender-stereotypic behaviors. Our results suggests that while behavior expectedness differs according to gender-typicality, judgments of behavior desirability are largely consistent for typical men and women (Studies 1a-1b), Black and Asian men and women (Studies 2a-2b), and successful working men and women (Studies 3a-3b). These results suggest that what is counter-stereotypic is not inherently undesirable and support the application of both expectancy violation theory and the unified theory of gender norm violations to predict reactions to gender norm violations.

Keywords: Gender stereotypes, gender bias, gender norms, racial stereotypes

Is what's unexpected undesirable? Investigating the expectedness and desirability of gender-stereotypic behaviors

In research on gender stereotypes and norms, we generally consider agency and communion to encompass masculine and feminine stereotypes, respectively. This is supported by decades of research that suggest agency and communion perceptions are the prime culprits for differences in our perceptions of men and women [1–4]. These stereotypes are thought to be not only descriptive but also prescriptive (gendered "shoulds") and proscriptive (gendered "should nots"), such that individuals think that men should engage in masculine behaviors and women should engage in feminine behaviors, and that men should not engage in feminine behaviors and women should not engage in masculine behaviors [5,6].

When individuals step out of their prescribed box, they are at risk of both social and economic penalties for counter-stereotypic behaviors [7–9]. For example, agentic and self-promoting women are rated as less likable and less hirable compared to equally qualified, agentic, and self-promoting men [10–12]. There is also evidence that women, but not men, are penalized for engaging in assertive, agentic leadership styles, attempting to exert influence through dominance, or simply succeeding at male-typed tasks [13–16]. While most research on this issue focuses on penalties for women, we see that men, too, are penalized for engaging in feminine-stereotypic behaviors such as self-effacing promotion styles, speaking modestly about past accomplishments, displaying sadness, asking for help, taking family leave, or succeeding in feminized careers such as education [10,17–21].

However, while we frequently see penalties for counter-stereotypic behaviors, these penalties do not always occur—for example, providing evidence of communion can ameliorate penalties for female managers [16], and women who are assigned to leadership roles (rather than seeking them out themselves) are not penalized for their leadership behaviors [22], while women who demonstrate high competence in masculine jobs are sometimes rated more highly than equally qualified men [23]. Additionally, there is growing evidence that women may be rewarded for acting agentically within organizations [24–26]. There is also evidence that men are not unequivocally punished for feminine-stereotypic behaviors: for example, communal men have been rated as equally or more hirable

than communal women [27], men who engage in helping behaviors in the workplace are rated more highly than women [28], and men engaging in transformational leadership styles are rated as more promotable than women due to increased communion perceptions [29]. There is even evidence that men who opt to take paternity leave after having children are recommended more highly for rewards and hiring than men who do not, again due to increased communion perceptions [30].

Predominant explanations for reactions to counter-stereotypic men and women

Role congruity theories

The lack of fit model [31,32] and role congruity theory [33] posit that penalties for counter-stereotypic behaviors occur because individuals violate prescriptive gender norms. Thus, individuals who engage in counter-stereotypic behaviors are sanctioned for perceived violations of their prescribed social roles [34]. These theories make the assumption that counter-stereotypic behaviors are inherently undesirable and predict that perceivers will penalize women who engage in masculine behaviors, and men who engage in feminine behaviors. They do not, however, account for rewards for counter-stereotypic behaviors.

Expectancy violation theory

Though it does not specifically deal with gender, expectancy violation theory can help explain the occurrence of rewards as well as penalties for counter-stereotypic behavior. Key to understanding its relevance is the idea that male and female stereotypes have positive and negative components—something often not attended to in the gender norm violation literature. That is, some counter-stereotypic behavior is positive in valence and other counter-stereotypic behavior is negative in valence (see Table 1). Expectancy violation theory posits that when individuals deviate from expectations, evaluations will become more extreme in the direction of the deviation, producing rewards for positive behavioral deviations and penalties for negative behavioral deviations [35]. Therefore, unlike the lack of fit model and role congruity theory, expectancy violation theory does not assume that what is unexpected is also undesirable; rather, an implicit assumption of expectancy violation theory is that behavior desirability is constant across targets.

Table 1

Positive and Negative, Masculine and Feminine Stereotypes

Valence	Masculine	Feminine
Positive	- Assertive	- Warm
	- Ambitious	- Friendly
	- Self-reliant	- Cooperative
	- Rational	- Patient
	- Decisive	- Sensitive
	- Athletic	- Cheerful
Negative	- Aggressive	- Emotional
	- Controlling	- Yielding
	- Stubborn	- Weak
	- Dominating	- Naïve
	- Arrogant	- Gullible
	- Angry	- Indecisive

Note. Positive masculine stereotypes are intensified prescriptions for men, positive feminine stereotypes are intensified prescriptions for women, negative masculine stereotypes are intensified proscriptions for women, and negative feminine stereotypes are intensified proscriptions for women [5,6].

If behavior desirability were determined entirely by stereotype consistency, then expectancy violation theory would add little to explaining reactions to counter-stereotypical behavior – if behaviors that are counter-stereotypic are inherently undesirable, expectancy violations would induce only penalties, not rewards. But if behavior gender-typicality and desirability are largely independent of one another, expectancy violation theory can help predict both positive and negative reactions to unexpected behavior. It would predict that if an individual acts in a way that is unexpected and desirable, they will be rewarded, and if an individual acts in a way that is unexpected and undesirable, they will be penalized.

A unified theory of gender norm violations

In Eareckson and Heilman [36], we develop a unified theory of gender norm violations that integrates both role congruity theories and expectation violation theory to explain the observed patterns of reactions to counter-stereotypic men and women. The unified model proposes that the assumptions of expectancy violation theory are generally accurate and can be applied to predict reactions to gender norm violations. However, it adds to the traditional expectancy violation model the moderating effect of gender congruence on desirability, such that individuals may perceive gender-congruent behaviors as more

desirable than gender-incongruent behaviors, in line with the predictions of role congruity theories [32,33]. In other words, we argue that there are general social norms that determine which behaviors are desirable or undesirable, but that gender congruity moderates the degree of desirability for these behaviors. More specifically, this theory suggests that although absolute social desirability is largely consistent across gender, there may be differences in relative desirability, such that masculine behaviors may be perceived as relatively more desirable in men than in women, and feminine behaviors may be perceived as relatively more desirable in women than in men.

The critical distinction between absolute desirability and relative desirability

We argue that knowledge of the absolute desirability of a behavior—judgments about whether the behavior is desirable or undesirable—is essential for predicting the direction of reactions to counter-stereotypic individuals. If a behavior is judged to be desirable in one gender but undesirable in the other gender, then reactions to men and women exhibiting the same behavior will differ. If, however, as we are proposing, absolute desirability is consistent between men and women, the direction of the reaction to a man or a woman engaging in this behavior will be consistent—i.e., a behavior that is desirable will receive favorable reactions, while a behavior that is undesirable will receive negative reactions regardless of the gender of the individual engaging in the behavior [36]. So absolute desirability is critical to understanding the direction of reactions to counter-stereotypic behavior.

However, the relative desirability of a behavior is not without consequence; a behavior that is absolutely desirable, but nonetheless *less* desirable in one gender than the other, would likely result in a less intense positive reaction to that individual while a behavior that is absolutely undesirable, but nonetheless *more* undesirable in one gender than the other, would likely result in a more intense negative reaction to that individual. Although they should not have an effect on the general direction of the outcome, relative differences in desirability will affect the magnitude of the positive or negative reaction. In other words, relative (but not absolute) differences in behavior desirability may reduce the rewards of a positive expectancy violation, and exacerbate the penalty of a negative expectancy violation, while differences in absolute desirability may result in the same behavior being rewarded in one gender, and

penalized in the other. See Fig. 1 for a visualization of how differences in behavior desirability would affect reactions to counter-stereotypic behaviors, under the predictions of the unified model.

For differences in expectedness, the distinction between absolute and relative expectedness is not so crucial. According to expectancy violation theory, the degree of expectedness determines how extreme a reaction will be, but not the overall direction of the reaction – that is determined by the absolute desirability, such that a desirable behavior will elicit a positive reaction, while an undesirable behavior will elicit a negative reaction. Assuming constant behavior desirability, it follows that a behavior that is absolutely unexpected will result in the largest reaction, and as the behavior becomes more expected, the magnitude of the reaction will shrink - however, the midpoint is not particularly meaningful; rather, it is the endpoint that is meaningful, when the behavior is absolutely expected; at this point, the unified model would predict no reaction whatsoever, since the behavior is entirely consistent with the existing perception of the individual. A behavior that is completely unexpected will elicit a larger reaction than a behavior that is somewhat unexpected, which will elicit a larger reaction than a behavior that is somewhat expected, which will in turn elicit a larger reaction than a behavior that is completely expected. Regardless of the strength of the reaction, though, it is absolute desirability, not expectedness, that determines whether the reaction is positive or negative. See Fig. 1 for a visualization of how expectedness differences should affect reactions to behaviors.

Fig. 1

Visualization of Predicted Reactions to Behaviors Varying in Expectedness and Desirability

Note: A reaction above 0 is indicative of a reward (positive improvement in perception), while a reaction below 0 is indicative of a penalty (negative improvement in perception).

The three theories we have discussed make competing predictions about the relationship between gender-typicality and behavior desirability – for gender congruity theories, gender-typicality should determine absolute desirability; for expectancy violation theory, gender-typicality should have no influence on desirability; and for the unified theory, gender-typicality should affect relative, but not

absolute, desirability. Thus, we can use evidence of studies investigating the desirability of gender-typical behaviors in men and women to evaluate the assumptions of these theories.

Existing evidence of the relationship between gender-typicality and desirability

Past studies investigating perceptions of gender-typicality and desirability have found that what is more typical of one gender than the other is also more desirable in that same gender [5,6,37]. On the face of it, these studies seem to support role congruity theories, such that traits are viewed as more desirable when they are gender-consistent and less desirable when they are gender-inconsistent. However, while these studies are useful and informative for understanding the content of descriptive stereotypes and prescriptive norms for men and women, they are not ideally suited to evaluate the assumptions of the unified theory of gender norm violations. This is because they focus on relative differences in desirability for men and women rather than the absolute desirability differences that are of central concern to us. While focusing on relative desirability is appropriate to the aim of identifying gender-based prescriptions and proscriptions of differing strength, it can be somewhat misleading when trying to predict positive and negative reactions to counter-stereotypic behavior.

For example, Prentice and Carranza [5] label the trait "friendly" as an intensified prescription for women and a relaxed prescription for men, despite the fact that "friendly" was actually rated as highly desirable in both women and men, with ratings of 8.30 and 7.32, respectively, on a scale ranging from 1 to 9. Indeed, "friendly" was rated as more desirable in men than four of the 'intensified prescriptions' for men—consistent (desirability of 7.24 in men), aggressive (desirability of 6.98 in men), intense (desirability of 6.81 in men), and forceful (desirability of 6.58 in men). The reason that consistent, aggressive, intense, and forceful are identified as intensified prescriptions for men, while happy (desirability of 7.49 in men), friendly (desirability of 7.32 in men), helpful (desirability of 7.12 in men), and clean (desirability of 7.05 in men) are not, is because the degree of prescription is identified by relative differences in desirability for men and women, as opposed to absolute desirability ratings. We argue that it is the absolute desirability ratings, not the relative ratings, that are crucial for predicting reactions to counter-stereotypic men and women.

An additional complication is that past studies measure trait typicality, rather than expectedness directly. Though it is probable that typicality and expectedness overlap, it is also possible that stereotype expectedness differs in some way from stereotype typicality. Another methodological issue is that past research primarily investigates traits and not behaviors– e.g. men are perceived as more assertive, ambitious, and independent than women, while women are perceived as more warm, friendly, and cooperative than men [5]; these personality traits are likely to be far more ambiguously valenced than actual behaviors. For example, a trait like 'assertive' could imply positive behaviors, such as taking charge in a group setting, or negative behaviors, such as interrupting another person when they are speaking. It may be that perceptions of specific behaviors differ from perceptions of broad character traits, which are inherently more ambiguous than behaviors. In the following research we address these issues by focusing on absolute behavior desirability in men and women, investigating perceptions of a set of gender-stereotypic behaviors rather than traits, and measuring expectedness directly to more explicitly examine the assumptions of the unified theory of gender norm violations.

Potential variations in expectedness and desirability

Intersectionality between race and gender stereotypes

Historically, research investigating perceptions of gender has been conducted with primarily White samples and focused on gender stereotypes and norms regarding White individuals. However, we know that intersectional identities can alter stereotypes, norms, and reactions to counter-stereotypic actors because individuals hold unique and complex stereotypes about individuals of different genders and races [38–40]. There is evidence, for example, that racial stereotypes are gendered, such that stereotypes about Asian individuals are more consistent with feminine stereotypes than masculine stereotypes, while stereotypes about Black individuals are more consistent with masculine stereotypes than feminine stereotypes [41].

The White male norm hypothesis [42] suggests that when race is not explicitly considered, participants are likely to assume and apply social default characteristics to study targets. Thus, when race is not explicitly mentioned in studies with predominantly White samples (the default in nationally

representative U.S. samples, as well as for typical Amazon MTurk and Prolific samples), we cannot assume that these results will generalize to perceptions of non-White targets. To understand how the ideas discussed here can be applied to men and women generally, it is therefore critical to study counter-stereotypic behavior not just in 'typical' men and women, who are likely to be perceived as White in prototypical U.S. samples, but also in non-White men and women, for whom behavior expectedness and desirability perceptions may differ.

Gender stereotypes and norms in the workplace

Although our understanding of gender stereotypes and norms is primarily based on investigations of prototypical men and women in society, many investigations of reactions to counter-stereotypic men and women occur in the workplace setting. However, it is possible that stereotypes and norms for successful working men and women differ from stereotypes and norms for typical men and women. Workplace expectations and requirements in the U.S. are generally centered around men and masculinity norms, and expectations of individuals in workplaces may therefore differ from expectations of individuals in society more broadly—particularly for women, since feminine stereotypes are less aligned with workplace norms than masculine stereotypes [43,44]. To better understand if the theories discussed here can be applied to understanding reactions to counter-stereotypical men and women in the workplace, it is important to examine behavior expectedness and desirability for successful working men and women, as behavior expectedness and desirability for successful working men and women may differ from the expectedness and desirability ratings for typical men and women.

The current research

In the six studies presented here, we investigate the expectedness and desirability of a set of twenty behaviors for men and women, varying in gender-typicality (masculine and feminine) and valence (positive and negative). We examine perceptions of behavior expectedness and desirability for 'typical' men and women (Studies 1a and 1b), 'typical' Black and Asian men and women (Studies 2a and 2b), and successful working men and women (Studies 3a and 3b). We had three primary goals in pursuing this program of research: (1) to validate the assumptions of the unified theory of gender norm violations by

examining the expectedness and desirability of a set of gender-stereotypic behaviors in typical men and women; (2) to determine whether our findings generalize to non-White targets by focusing on Black and Asian men and women; and (3) to determine whether our findings generalize to the work context by focusing on successful working men and women.

Hypotheses

Hypothesis 1: Behaviors will differ in relative expectedness, such that behaviors are rated as more expected in the gender they are stereotypic in. If we see a significant interaction between target gender and behavior gender-typicality in Studies 1a, 2a, and 3a, such that feminine-stereotypic behaviors are more expected of women than men and masculine-stereotypic behaviors are more expected of men than women, this provides evidence that behaviors are relatively more expected in the gender they are stereotypic of.

Hypothesis 2: Behaviors will be consistent in absolute desirability, regardless of gender-typicality. If positive behaviors are rated as desirable in both men and women (95% CIs above 0) and negative behaviors are undesirable in both men and women (95% CIs below 0) in Studies 1b, 2b, and 3b, these results will support our hypothesis.

Hypothesis 3: Behaviors will differ in relative desirability, such that behaviors are rated as more desirable in the gender they are stereotypic in. If we see a significant interaction between target gender and behavior gender-typicality in Studies 1b, 2b, and 3b, such that feminine-stereotypic behaviors are more desirable in women than men and masculine-stereotypic behaviors are more desirable in men than women, this provides evidence that behaviors are relatively more expected in the gender they are stereotypic of. This finding would be consistent with past research on stereotypes and norms for men and women [5.6.37].

Methods

Procedure

Participants consented to the survey and were asked to answer the survey questions honestly and pay attention to each behavior they were asked to respond to. They then were presented with twenty

stimulus behaviors and instructed to rate the expectedness of each behavior for their assigned target by answering the question: "How [expected/desirable] do you think this behavior is for a [TARGET]?" Participants rated each of the twenty behaviors on a seven-point scale from 'extremely [unexpected/undesirable]' to 'extremely [expected/desirable]'. Participants were randomly assigned to one of the possible targets in each study and evaluated all twenty behaviors in Table 2 in randomized order. Data collection for these studies began on 02/18/2021 and was completed on 04/16/2022.

Table 2

Behaviors Investigated in Studies 1a-3b

Category	Behavior
Masculine Positive	- Takes charge in group settings<br>- Negotiates for the best deal on products and services [*Negotiates for higher raises and bonuses*]<br>- Speaks positively about their accomplishments<br>- Seeks leadership roles [*Seeks opportunities for advancement*]<br>- Plays to win in games and sports [*Competes with co-workers for high profile assignments*]
Masculine Negative	- Often interrupts or speaks over others when they are talking<br>- Never admits they are wrong<br>- Brags about their accomplishments<br>- Loses their temper when frustrated<br>- Never asks for or accepts help on projects
Feminine Positive	- Goes out of their way to help others<br>- Anticipates and takes care of others' needs<br>- Maintains a positive attitude<br>- Takes care of household chores [*Offers to take notes in meetings*]<br>- Plans parties for friends and family [*Plans parties for co-workers' birthdays*]
Feminine Negative	- Apologizes often, even when they have done nothing wrong<br>- Always backs down from confrontations<br>- Cries easily when frustrated<br>- Is sensitive and their feelings are easily hurt<br>- Lets others interrupt them and speak over them

Note: Behavior modifications for Studies 3a-3b to be consistent with the successful working role are indicated in brackets.

In developing the set of 20 behaviors, we utilized the trait stereotypes and norms identified by past research on descriptive gender stereotypes and prescriptive norms [5,6] as the foundation for generating five masculine positive (e.g. takes charge in group settings), five masculine negative (e.g. brags about their accomplishments), five feminine positive (e.g. goes out of their way to help others), and five feminine negative (e.g. always backs down from confrontations) behaviors. We pilot tested these behaviors with 100 Amazon MTurk raters. Participants were asked to classify each of the twenty behaviors as stereotypically masculine or stereotypically feminine, and as positive or negative; we tested each behavior categorization with chi square goodness of fit tests comparing the observed distributions to the 50/50 distributions that would be consistent with chance.

This pilot test confirmed that participant perceptions of all twenty behaviors were consistent with our theoretical classifications, with all ten masculine behaviors classified as significantly more masculine than feminine (min = interrupts others at 72% masculine, max = plays to win at 92% masculine), all ten feminine behaviors classified as significantly more feminine than masculine (min = maintains positive attitude at 61% feminine, max = cries easily at 91% feminine), all ten positive behaviors classified as significantly more positive than negative (min = plays to win at 83% positive, max = maintains positive attitude at 97% positive), and all ten negative behaviors classified as significantly more negative than positive (min = backs down from confrontations at 71% negative, max = interrupts others at 90% negative). These results indicate that all twenty behaviors are effective manipulations of gender stereotypicality and valence in the intended direction.

Analyses. To test hypotheses 1 and 3, we fit linear mixed models for the effect of the interaction between target gender (effects coded, men = -1 and women = 1) and behavior gender-typicality (effects coded, masculine = -1 and feminine = 1) on expectedness and desirability, respectively, including random intercepts for participant ID and specific behavior. To test hypothesis 2, we analyzed the 95% confidence intervals on the behavior-level means to identify whether each positive behavior was viewed as desirable

for both men and women, and whether each negative behavior was viewed as undesirable for both men and women. Data were analyzed using RStudio 2023.09.1 for Windows. All data and analyses are available at https://osf.io/mpfsy/?view_only=89be74a03d9a4decacb03c5143b62a5d

Study 1a

In Study 1a, we investigate how expected gender-stereotypic behaviors are for typical men and women, answering the question "How expected do you think this behavior is for a TYPICAL [MAN/WOMAN]?" on a seven-point scale ranging from 'completely unexpected' (0) to 'completely expected' (6). Our key prediction was that behaviors would be relatively more expected for the gender they are stereotypic of (hypothesis 1).

Participants

103 Amazon MTurk workers were recruited from CloudResearch and participated in the study online for $0.40 monetary compensation; 3 participants did not respond to one or more of the key dependent variables and were excluded from the analyses. Of the remaining 100 participants, 40 identified as male (40%) and 58 identified as female (58%); 2 participants identified as a gender other than male or female or chose not to disclose their gender identification (2%). 71 participants identified as White (71%), 11 as Asian (11%), 8 as Hispanic (8%), and 6 as Black (6%); 4 participants identified as another ethnicity or declined to identify (4%). Participants' ages ranged from 18 to 68 with a median age of 32.

Results

Hypothesis 1: Relative differences in expectedness by gender

As predicted in Hypothesis 1, there was a significant two-way interaction between target gender and behavior gender-typicality, $b = .18$, $t(3666) = 8.48$, $p < .001$, such that feminine-stereotypic behaviors were more expected of women ($M = 4.16$, $SD = 1.35$) than men ($M = 2.22$, $SD = 1.51$), and masculine-stereotypic behaviors were more expected of men ($M = 4.18$, $SD = 1.25$) than women ($M = 3.04$, $SD = 1.54$). See Fig. 2 for a visualization of this interaction. We saw large effect sizes for each item,

ranging from d = .46 for losing one's temper to d = 2.73 for crying when frustrated; the average effect size across all twenty behaviors was d = 1.27. See Fig. 3 for the behavior-level expectedness ratings.

Fig. 2

The interaction between behavior gender-typicality and target gender in ratings of behavior expectedness for typical men and women

Fig. 3

Behavior-level expectedness means with 95% CIs for typical men and women

Summary

Behaviors were more expected in the gender they are stereotypic of (hypothesis 1 supported). We see large effect sizes for the difference in expectedness for all items studied, suggesting that gender has a very large influence on the expectedness of the twenty behaviors studied here for typical men and women.

Study 1b

In Study 1b, we investigate how desirable the set of gender-stereotypic behaviors examined in Study 1a are for typical men and women. The study had the same design and procedure as Study 1a, except that participants rated how desirable (not expected) each of the twenty behaviors were on a seven-point scale from 'extremely undesirable' (-3) to 'extremely desirable' (3); the value of 0 corresponds to the scale midpoint, or the point at which the behavior is perceived as neither desirable nor undesirable. Our key prediction was that behaviors would be rated consistently in absolute desirability for both men and women (hypothesis 2); values below 0 indicate that a behavior was rated as undesirable, and values above 0 indicate that a behavior was rated as desirable. We also predicted that behaviors would differ in relative desirability for men and women, such that masculine behaviors are relatively more desirable in men than in women, and feminine behaviors are relatively more desirable in women than men (hypothesis 3).

Participants

102 Amazon MTurk workers participated in the study online for $0.40 monetary compensation; 2 participants did not respond to one or more of the key dependent variables and were excluded from the

analyses. Of the remaining 100 participants, 51 identified as male (51%) and 47 identified as female (47%); 2 participants identified as a gender other than male or female or chose not to disclose their gender identification (2%). 77 participants identified as White (77%), 7 as Black (7%), 6 as Asian (6%), and 5 as Hispanic (5%); 5 participants identified as another ethnicity or declined to identify (5%). Participants' ages ranged from 20 to 73 with a median age of 32.

Results

Hypothesis 2: Absolute consistency in desirability

For both male and female targets, we saw consistent absolute desirability ratings across all twenty behaviors studied – all five positive masculine-stereotypic behaviors and all five positive feminine-stereotypic behaviors were seen as desirable in both men and women (95% CIs above 0), while all five negative masculine-stereotypic behaviors and all five negative feminine-stereotypic behaviors are seen as undesirable in both men and women (95% CIs below 0, see Fig. 4 for a visualization).

Fig. 4

Behavior-level desirability means with 95% CIs for typical men and women

Hypothesis 3: Relative differences in desirability by gender

Surprisingly, we did not find an interaction between target gender and behavior gender-typicality, $b < .01$, $t(2827) = 1.24$, $p = .216$, indicating that relative behavior desirability did not differ according to behavioral gender consistency (see Fig. 5).

Fig. 5

The interaction between behavior gender-typicality and target gender in ratings of behavior desirability for typical men and women

Summary

Consistent with our predictions, we found that positive behaviors were rated as absolutely desirable in both men and women and negative behaviors were rated as absolutely undesirable in both men and women, regardless of gender stereotypicality (hypothesis 2 supported). Inconsistent with our predictions, there was no interaction between target gender and behavior gender-typicality on the

desirability ratings, suggesting that not only was absolute desirability consistent between men and women, but relative desirability was consistent as well (hypothesis 3 not supported).

Study 2a

In Study 2a, we investigate how expected a set of gender-stereotypic behaviors are for typical Asian and typical Black men and women, to examine whether our results from Study 1a are generalizable to perceptions of non-White individuals. The design and methods were identical to Study 1a, except that we manipulated target race, as well as target gender. Participants answered the question: "How expected do you think this behavior is for a TYPICAL [BLACK/ASIAN] [MAN/WOMAN]?" Our key prediction was that behaviors would be relatively more expected for the gender they are stereotypic of, and that would be so regardless of the target's race. Additionally, we examine the effects of race on behavior expectation. Consistent with our analyses of the gender and behavior gender-typicality variables, race was effects coded before being added to the model (Asian = -1, Black = 1).

Participants

205 Amazon MTurk workers participated in the study online for $0.40 monetary compensation; 4 participants did not respond to one or more of the key dependent variables and were excluded from the analyses. Of the remaining 201 participants, 70 identified as male (34.83%) and 126 identified as female (62.69%); 5 participants identified as a gender other than male or female or chose not to disclose their gender identification (2.49%). 101 participants identified as White (50.25%), 41 as Black (20.40%), 27 as Hispanic (13.43%), and 18 as Asian (8.96%); 14 participants identified as another ethnicity or declined to identify (6.97%). Participants' ages ranged from 18 to 72 with a median age of 34.

Results

Hypothesis 1: Relative differences in expectedness by gender

As predicted, there was a significant interaction between target gender and behavior gender-typicality, $b = .17$, $t(3797) = 7.75$, $p < .001$, such that masculine-stereotypic behaviors were more expected of men ($M = 3.40$, $SD = 1.50$) than women ($M = 3.27$, $SD = 1.60$) and feminine-stereotypic behaviors were more expected of women ($M = 3.43$, $SD = 1.60$) than men ($M = 2.87$, $SD = 1.59$).

However, we also saw a significant three-way interaction between target gender, target race, and behavior gender-typicality, $b = -.06$, $t(3797) = 2.73$, $p = .006$. When we decompose this by looking at the two-way interaction between target gender and behavior gender-typicality for both Asian targets, $b = .23$, $t(1861) = 7.08$, $p < .001$, and Black targets, $b = .11$, $t(1918) = 3.90$, $p < .001$, we see that the two-way interaction between target gender and behavior gender-typicality is significant for both Asian and Black men and women; however, the interaction is larger for Asian targets, relative to Black targets. This seems to be driven by a higher expectation of masculine behaviors in Asian men ($M = 3.30$, $SD = 1.56$) than in Asian women ($M = 2.91$, $SD = 1.73$), but similar expectations of masculine behaviors in Black men ($M = 3.50$, $SD = 1.33$) and in Black women ($M = 3.64$, $SD = 1.37$). See Fig. 6 for a visualization. Behavior-level effect sizes ranged from d = .02 (helps others) to d = 1.21 (does household chores); the mean effect size was small-medium, d = .37. See Fig. 7 for a visualization of the behavior-level means.

Fig. 6

The interaction between behavior gender-typicality and target gender in ratings of behavior expectedness for typical Asian and Black men and women

Fig. 7

Behavior-level expectedness means with 95% CIs for typical Asian and Black men and women

Relative differences in behavior expectedness by race

There was a significant interaction between target race and behavior gender-typicality, $b = -.20$, $t(3797) = 9.14$, $p < .001$, such that masculine-stereotypic behaviors were more expected of typical Black individuals ($M = 3.56$, $SD = 1.35$) than of typical Asian individuals ($M = 3.09$, $SD = 1.66$), and feminine-stereotypic behaviors were more expected of typical Asian individuals ($M = 3.33$, $SD = 1.59$) than typical Black individuals ($M = 2.99$, $SD = 1.63$). After decomposing the significant three-way interaction, this interaction remains significant for both men, $b = -.14$, $t(1861) = 4.57$, $p < .001$, and women, $b = -.26$, $t(1918) = -8.41$, $p < .001$, though it is stronger for women than for men. Effect sizes varied from d = .02 (anticipates others' needs) to 1.20 (interrupts others when talking), with a medium average effect size of d = .45.

Summary

Behaviors were relatively more expected in the gender they are stereotypic of (hypothesis 1 supported); however, a significant three-way interaction between target gender, target race, and behavior gender-typicality suggests that this effect is stronger for Asian men and women than for Black men and women. In particular, it is notable that the condition means for the expectedness of masculine behaviors for Black men and women were extremely similar, suggesting that the expectedness differences we observe for masculine behaviors hold for prototypical men and women and for Asian men and women, but may not generalize to Black men and women. Additionally, we saw smaller effect sizes on the behavior-level differences in expectedness for Asian and Black men and women, compared to in Study 1a. Altogether, these results suggest that our conclusions from Study 1a partially generalize to typical Asian and Black men and women, although information about racial identity may weaken the effect of target gender on behavioral expectations, since we saw smaller effect sizes of gender on behavior expectedness in this study, compared to in Study 1a.

In addition to the effects of target gender, we found evidence for an interaction between target race and gender on behavior expectedness, such that feminine behaviors were more expected of Asian targets than of Black targets, and masculine behaviors were more expected of Black targets than of Asian targets. This is consistent with past work suggesting that race is gendered, such that Asian individuals are stereotyped as feminine, and Black individuals are stereotyped as masculine [45,46].

Study 2b

In Study 2b, we investigate how desirable the set of gender-stereotypic behaviors examined in Study 2a are for Asian and Black men and women; we used the same design and procedure as in Study 2a, except that participants evaluated how desirable the behaviors were, rather than how expected. Our key predictions were behaviors would be consistent in absolute desirability for men and women (hypothesis 2), and that behaviors would differ in relative desirability for men and women, regardless of race and gender, although we did not see this pattern in Study 1b (hypothesis 3). As in Study 2a, we also examine the effects of race on desirability.

Participants

203 Amazon MTurk workers participated in the study online for $0.40 monetary compensation; 4 participants did not respond to one or more of the key dependent variables and were excluded from the analyses. Of the remaining 199 participants, 78 identified as male (39.20%) and 117 identified as female (58.79%); 4 participants identified as a gender other than male or female or chose not to disclose their gender identification (2.01%). 105 participants identified as White (52.76%), 39 as Black (19.60%), 24 as Hispanic (12.06%), and 22 as Asian (11.06%); 9 participants identified as another ethnicity or declined to identify (4.52%). Participants' ages ranged from 18 to 70 with a median age of 33.

Results

Hypothesis 2: Absolute consistency in desirability

We saw consistent absolute desirability for Asian men and women across nineteen of the twenty behaviors, with all five positive feminine behaviors and all five positive masculine behaviors seen as absolutely desirable for both Asian men and Asian women (95% CIs above 0), and all five negative feminine behaviors seen as absolutely undesirable for both Asian men and Asian women (95% CIs below 0). We saw four out of five negative masculine behaviors rated as absolutely undesirable for both Asian men and Asian women (95% CIs below 0); the only exception was "never asking for help," which was rated as absolutely undesirable for Asian women (95% CI [-.78 -.17]), but neither absolutely desirable nor undesirable for Asian men (95% CI [-.65 .17]).

We observed consistent absolute desirability for all twenty behaviors for Black men and women, with all five positive feminine behaviors and all five positive masculine behaviors seen as absolutely desirable for both Black men and Black women (95% CIs above 0), and all five negative feminine behaviors seen as absolutely undesirable for both Black men and black women (95% CIs below 0). Again, we saw four out of five negative masculine behaviors rated as absolutely undesirable for both Black men and Black women (95% CIs below 0); the only exception was "brags about accomplishments," with the 95% CI including 0 for both Black men (95% CI [-.92 .04]) and Black women (95% CI [-.69 .14]). See Fig. 8 for a graph of the behavior-level means.

Fig. 8

Behavior-level desirability means for typical Asian and Black men and women

Hypothesis 3: Relative differences in desirability by gender

We found a significant interaction between target gender and behavior gender-typicality, $b = .05$, $t(3759) = 2.30$, $p = .021$, indicating that masculine-stereotypic behaviors were relatively more desirable in men ($M = .13$, $SD = 1.96$) than in women ($M = .08$, $SD = 1.89$), while feminine-stereotypic behaviors were relatively more desirable in women ($M = .42$, $SD = 1.87$) than in men ($M = .29$, $SD = 1.94$). However, this was qualified by a significant three-way interaction between target race, target gender, and behavior gender-typicality, $b = -.08$, $t(3759) = -3.61$, $p < .001$, such that the two-way interaction between target gender and behavior gender-typicality was significant for Asian targets, $b = .13$, $t(1842) = 4.41$, $p < .001$, but not Black targets, $b = -.03$, $t(1899) = -.89$, $p = .375$. This suggests that feminine behaviors were relatively more desirable in Asian women ($M = .61$, $SD = 1.76$) than Asian men ($M = .28$, $SD = 181$), and masculine behaviors were relatively more desirable in Asian men ($M = .07$, $SD = 1.87$) than Asian women ($M = -.12$, $SD = 1.81$), but that we saw similar desirability of feminine behaviors for Black women ($M = .24$, $SD = 1.94$) and Black men ($M = .19$, $SD = 2.06$), as well as of masculine behaviors for Black men ($M = .29$, $SD = 2.06$) and Black women ($M = .26$, $SD = 1.94$). We observed variable effect sizes across behaviors for the desirability difference between Asian men and women, ranging from d < .01 (apologizes often) to d = .62 (cries when frustrated), with an average effect size of d = .26. For Black men and women, we observed effect sizes ranging from d = .02 (never admits when they are wrong) to d = .50 (cries when frustrated), with an average effect size of d = .19. See Fig. 9 for a visualization of the interactions.

Fig. 9

The interaction between behavior gender-typicality and target gender in ratings of behavior desirability for typical Asian and Black men and women

Relative differences in desirability by race

We found a significant interaction between target race and behavior gender-typicality, $b = -.11$, t(3759) = 4.99, $p < .001$, such that masculine behaviors were rated as relatively more desirable in Black ($M = .23$, $SD = 1.98$) than Asian ($M = -.02$, $SD = 1.84$) targets, while feminine behaviors were rated as relatively more desirable in Asian ($M = .44$, $SD = 1.80$) than Black ($M = .26$, $SD = 2.00$) targets. However, when decomposing the three-way interaction between target race, target gender, and behavior gender-typicality, we find that the interaction between target race and behavior gender-typicality was significant only for women, $b = -.19$, $t(1861) = 5.99$, $p < .001$, but not for men, $b = -.03$, $t(1880) = 1.00$, $p = .318$. This suggests that in particular, we see that feminine behaviors were more relatively desirable in Asian women ($M = .61$, $SD = 1.76$) than in Black women ($M = .24$, $SD = 1.94$), and masculine behaviors were relatively more desirable in Black women ($M = .26$, $SD = 1.94$) than in Asian women ($M = -.12$, $SD = 1.81$), but that we saw similar desirability of feminine behaviors for Black men ($M = .19$, $SD = 2.06$) and Asian men ($M = .28$, $SD = 181$), and of masculine behaviors for Black men ($M = .29$, $SD = 2.06$) and Asian men ($M = .07$, $SD = 1.87$).

Summary

Consistent with Study 1b, we found that absolute behavior desirability was largely consistent for both Black and Asian men and women; the only exception was "never asks for or accepts help on projects," which was absolutely undesirable in Asian men, but neither undesirable nor desirable in Asian women (hypothesis 1 largely supported). Additionally, we saw that for both Black men and women, the behavior "brags about their accomplishments" was neither undesirable nor desirable, as the confidence interval included zero. Unlike in Study 1b, we found an interaction between target gender and behavior gender-typicality on desirability, such that behaviors were rated as more desirable in the gender they are stereotypic of; however, this was qualified by a three-way interaction, indicating that the interaction was driven by differences in behavior desirability for Asian men and women, but not for Black men and women (hypothesis 3 partially supported). Overall, the result patterns for absolute desirability are consistent with those of Study 1b, indicating that the absolute desirability of behaviors is largely

consistent for Asian men and women, as well as Black men and women; in addition, these results also provide some support for differences in relative desirability according to gender, particularly for Asian men and women.

Study 3a

In Study 3a, we investigate how expected a set of gender-stereotypic behaviors are for successful working men and women. The design was identical to Study 1a, except that participants were randomly assigned to evaluate the behaviors for a successful working man or successful working woman, rather than a typical man or typical woman. Our key prediction was that behaviors would be relatively more expected in the gender they are stereotypic of.

Participants

197 Amazon MTurk workers participated in the study online for $0.40 monetary compensation; 3 participants did not respond to one or more of the key dependent variables and were excluded from the analyses. Of the remaining 194 participants, 85 identified as male (43.81%) and 107 identified as female (55.15%); 2 participants identified as a gender other than male or female or chose not to disclose their gender identification (1.03%). 139 participants identified as White (71.65%), 19 as Hispanic (9.79%), 19 as Asian (9.79%), and 10 as Black (5.15%); 7 participants identified as another ethnicity or declined to identify (3.61%). Participants' ages ranged from 18 to 71 with a median age of 31.

Results

Hypothesis 1: Relative differences in expectedness

As predicted, there was a significant two-way interaction between target gender and behavior gender-typicality, $b = .18$, $t(3666) = 8.48$, $p < .001$, such that feminine-stereotypic behaviors were relatively more expected of successful working women ($M = 3.73$, $SD = 1.94$) than successful working men ($M = 3.50$, $SD = 1.96$), and masculine-stereotypic behaviors were relatively more expected of successful working men ($M = 4.84$, $SD = 1.89$) than successful working women ($M = 4.32$, $SD = 2.00$). See Fig. 10 for a visualization of this interaction. Effect sizes ranged from d < .01 (speaks positively about

accomplishments) to d = .64 (brags about accomplishments); the mean effect size across behaviors was d = .29. See Fig. 11 for a graph of the behavior-level means.

Fig. 10

The interaction between behavior gender-typicality and target gender in ratings of behavior expectedness for successful working men and women

Fig. 11

Behavior-level expectedness means with 95% CIs for successful working men and women

Summary

As in Studies 1a and 2a, there was a significant two-way interaction between target gender and behavior gender-typicality in the predicted direction, with behaviors rated as more expected in the gender they are stereotypic of (hypothesis 1 supported). However, the effect sizes on the differences in behavior desirability for men and women were much smaller for successful working targets, compared to typical men and women; the effect sizes in this study were even smaller than the effect sizes observed in Study 2b for typical Black and Asian men and women. This suggests that information indicating that an individual is successful in the workplace may result in smaller effects of gender on the expectedness of stereotypically masculine and feminine behaviors, compared to the effects of gender on expectedness in typical targets.

Study 3b

In Study 3b, we investigate how desirable the set of gender-stereotypic behaviors examined in Study 3a are for successful working men and women. The design and procedure were the same as in Study 1b, except that participants were randomly assigned to evaluate the behaviors for a successful working man or a successful working woman, rather than a typical man or typical woman. Our key prediction was that behaviors would be rated consistently in absolute desirability for both men and women. We also predicted that there would be relative differences in desirability according to target gender, although we did not observe this in Study 1b, and only observed this for Asian men and women in Study 2b.

Participants

199 Amazon MTurk workers participated in the study online for $0.40 monetary compensation; 2 participants did not respond to one or more of the key dependent variables and were excluded from the analyses. Of the remaining 197 participants, 78 identified as male (39.59%) and 113 identified as female (57.36%); 6 participants identified as a gender other than male or female or chose not to disclose their gender identification (3.05%). 149 participants identified as White (75.63%), 18 as Black (9.14%), 14 as Asian (7.12%), and 8 as Hispanic (4.06%); 8 participants identified as another ethnicity or declined to identify (4.06%). Participants' ages ranged from 18 to 78 with a median age of 31.

Results

Hypothesis 2: Absolute consistency in desirability

For both male and female targets, we saw consistent absolute desirability ratings across all twenty behaviors studied – all five positive masculine-stereotypic behaviors and all five positive feminine-stereotypic behaviors are seen as desirable in both men and women (95% CIs above 0), while all five negative masculine-stereotypic behaviors and all five negative feminine-stereotypic behaviors are seen as undesirable in both men and women (95% CIs below 0). See Fig. 12 for a visualization of the behavior-level means.

Fig. 12

Behavior-level desirability means with 95% CIs for successful working men and women

Hypothesis 3: Relative differences in desirability

We found a significant two-way interaction between target gender and behavior gender-typicality, $b = -.10$, $t(3723) = 5.00$, $p < .001$ in the opposite direction as we predicted, such that feminine-stereotypic behaviors were rated as relatively more desirable for men ($M = .18$, $SD = 2.38$) than for women ($M = -.07$, $SD = 2.35$) and masculine-stereotypic behaviors were rated as relatively more desirable for women ($M = .06$, $SD = 2.62$) than for men ($M = -.10$, $SD = 2.64$) (hypothesis 3 not supported). See Fig. 13 for a visualization of this interaction. The effect sizes on the individual behavior differences in desirability for

successful working men and women ranged from d < .01 (is sensitive and gets their feelings hurt) to d = .47 (takes notes in meetings); the mean effect size was d = .19.

Fig. 13

The interaction between behavior gender-typicality and target gender in ratings of behavior desirability for successful working men and women

Summary

As in Studies 1b and 2b, positive behaviors were rated as desirable in both male and female targets and negative behaviors were rated as undesirable in both male and female targets, regardless of behavior gender-typicality (hypothesis 2 supported). Unlike in Studies 1b and 2b, there was a significant two-way interaction between target gender and behavior gender-typicality, such that gender-stereotypic behaviors were seen as more desirable in the gender they are atypical of (hypothesis 3 not supported). This result is surprising and contradicts past research on stereotype desirability [5,6], which suggests that traits are more desirable in the gender they are stereotypic of, and it is unclear why this pattern occurred for successful working targets, but not for men and women in Study 1b or 2b. Overall, these results indicate that absolute behavior desirability is largely consistent for successful working men and women, although there may be small differences in the relative desirability of behaviors.

Discussion

Summary of results

Together, these results suggest that relative behavior expectedness differs for typical men and women, typical Black and Asian men and women, and successful working men and women, according to gender-typicality, while absolute behavior desirability is largely consistent, regardless of gender-typicality. Our results also suggest that differences in behavior expectedness are strongest for prototypical targets, and thus reactions to counter-stereotypic behaviors may be largest when researchers study gender norm violations in society, generally, compared to targets with more specific identifying information, such as information about racial identity or occupation. In Studies 2a and 3a, where we investigated behavior expectedness in typical Black and Asian targets and in successful working contexts,

we saw much smaller effect sizes for the differences in behavior expectedness according to gender compared to typical targets in Study 1a, suggesting that information about target race or work context may result in smaller expectancy violations for gender-inconsistent behaviors, and therefore rewards or penalties for counter-stereotypic behaviors may be weaker or less common in studies investigating reactions to Black or Asian men and women, or working men and women, compared to studies investigating reactions to prototypical men and women. This is consistent with work suggesting that White women are punished more severely for displaying dominant behaviors than Black women, as dominance is less stereotypical and therefore less expected of White women than of Black women [47].

However, we see very little support for our hypothesis that relative behavior desirability would differ between men and women. We did not see evidence that behaviors were more desirable in the gender they are stereotypic of for typical men and women, typical Black men and women, or successful working men and women - in fact, for successful working men and women, we saw evidence of the opposite effect, with behaviors rated as more desirable in the gender they are counter-stereotypic for. We only saw the predicted pattern for relative desirability in typical Asian men and women, with masculine behaviors rated as relatively more desirable in Asian men than in Asian women, and feminine behaviors rated as relatively more desirable in Asian women than in Asian men.

It is unclear why we did not see much evidence for relative desirability differences between men and women, such that behaviors are more desirable in the gender they are stereotypic of, or why we saw the opposite pattern in Study 3b for successful working men and women. One possibility is that by having participants evaluate behaviors instead of traits, as past work has done [5,6,37], we reduced the ambiguity of the ratings, resulting in more consistent desirability ratings for men and women. Another possibility is that society has changed in the decades since these studies were conducted, and that relative desirability differences in these behaviors are smaller than they used to be, or non-existent, or alternatively that social pressure to be egalitarian is stronger now than it used to be, resulting in a hesitance to show gender bias against gender-stereotypic men and women by giving low desirability ratings to men engaging in feminine-stereotypic behaviors, or to women engaging in masculine-stereotypic behaviors. The reversed

pattern in Study 3b is particularly surprising, and suggests that there may be something unique about the working context that makes it desirable for individuals to overcome gender stereotypes and norms and act in counter-stereotypic ways; however, future work must test whether the observed differences in relative desirability generalize to more specific work settings when individuals actually enact these types of behaviors.

One caveat to our lack of support for our third hypothesis is that there was quite a bit of variability in the effect sizes of relative desirability differences across behaviors - in particular, looking at the patterns of desirability ratings for behaviors across studies, we see that the behavior "cries easily when frustrated" was consistently rated as less desirable in men than in women across all targets. Thus, there are likely to be specific behaviors that are perceived as more desirable in the gender they are stereotypic of; however, our results suggest that this is not a general pattern across all gender-stereotypic behaviors. Therefore, it is important for researchers to consider whether or not desirability differs for men and women in the specific materials they plan to use, and for the specific targets they are interested in studying, to accurately predict reactions to counter-stereotypic men and women.

These results provide support for the implicit assumptions of expectancy violation theory and the unified theory- that behavior expectedness differs between men and women, but absolute desirability is consistent, and support the application of expectancy violation theory or the unified theory to predict reactions to counter-stereotypical men and women. They contradict past work suggesting that trait stereotypes broadly differ in relative desirability between men and women, and suggest that there may be situations where the unified model proposed by Eareckson and Heilman [36] can be simplified to the basic expectancy violation model, as gender may not always moderate behavior desirability. More work must be done to determine the contextual factors that result in weaker effects of gender role congruence on behavior desirability, and whether there are particular subsets of behaviors, such as crying when frustrated, that do show the predicted differences in relative desirability.

Limitations

All of our studies utilize American samples from Amazon MTurk, and it is therefore possible that these results may not generalize outside of this specific population. Another limitation of this work is that the participants in these studies are primarily White, and the sample demographics are not nationally representative. This is a particular issue for Studies 2a-2b, as it is possible that the patterns we are seeing are a result of racial stereotypes applied by outgroup members, and the gender stereotypes that exist for Asian and Black men and women within the ingroup may follow a different pattern.

In addition, the gender designation of our study targets and participants is based on a limited view of gender as a binary construct, when we know that gender identity truly exists as a continuum; our participants primarily identify as men or women, and we only collect data regarding perceptions of these behaviors in men and women. To gain a broader understanding of the patterns of behavior expectedness and desirability for men and women, it is important to replicate this work with nationally representative samples from the US and other countries, as well as examine perceptions of behavior expectedness in targets varying in identities other than race, and in contexts besides the workplace environment.

Additionally, although part of our purpose in defining behaviors so simply was to define behavior gender-typicality and valence, the behaviors studied here are a sanitized version of what these behaviors would be in real men and women, both in society and in the workplace. We chose to examine high-level perceptions of gender-stereotypic behaviors by asking participants to provide their impressions of behaviors with a one-line description. In reality, these behaviors take place in complex social settings and can be enacted in a variety of ways, which certainly will influence the perceived expectedness and desirability of the behavior. Future work should take into account these base-level perceptions of expectedness and desirability and investigate how more complex or ambiguous situations affect perceptions of behavior expectedness and desirability in men and women who violate gender norms.

Conclusions

Our results support the assumptions of expectancy violation theory over role congruity theories, supporting the application of expectancy violation theory in research on reactions to counter-stereotypic

men and women. Additionally, our results provide some support for the moderation of behavior desirability by gender proposed by the unified theory of gender norm violations, specifically for typical Asian men and women. Our results also suggest that considerations of identities beyond the labels 'typical man' and 'typical woman' are critical for understanding behavior expectedness, since we see smaller effect sizes for relative differences in behavior expectedness for typical Black, typical Asian, and successful working men and women, compared to prototypical men and women.

Our findings indicate that absolute behavior desirability depends on valence, not stereotypicality. These findings are particularly intriguing when considering positive aspects of agency which current work suggests are viewed negatively and penalized in women, while being essential for organizational success – for example, taking charge in group settings, negotiating, engaging in self-promotion, seeking out leadership roles, and competing for assignments. The strong consistency of the absolute desirability of positive behaviors for all targets, regardless of the expectancy ratings, can be used to encourage individuals to engage in counter-stereotypic behaviors. There is evidence that individuals often modify or restrict their behavior out of fear of backlash for violating gender norms; our results here, in conjunction with the predictions of expectancy violation theory, suggest that backlash is not inevitable when individuals engage in counter-stereotypic behaviors. These results can thus be used to assuage fears of backlash and encourage individuals to engage in positive, counter-stereotypic behaviors that can improve their quality of life or occupational and educational outcomes, such as encouraging women to negotiate for raises or seek leadership roles, and encouraging men to help others or take care of household chores.

The six studies presented here lend support to the assumptions of expectancy violation theory, rather than gender congruity theories, and validate the application of the unified theory of gender norm violations to study reactions to counter-stereotypic men and women. The behavior-level expectedness and desirability data provided in the OSF materials can also be used by researchers to develop manipulations and hypotheses for empirical experiments studying reactions to counter-stereotypic men and women, as these means provide critical information regarding current behavioral gender stereotypes and norms for prototypical, Black and Asian, and successful working men and women in the United States. We hope that

the insights from these studies can help advance both our theoretical and empirical understanding of why and when individuals may be penalized or rewarded for violating behavioral gender stereotypes, and inform the development of future studies exploring this topic.

References

1. Holt CL, Ellis JB. Assessing the current validity of the Bem Sex-Role Inventory. *Sex Roles.* 1998;39:929–41.
2. Haines EL, Deaux K, Lofaro N. The times they are a-changing… or are they not? A comparison of gender stereotypes, 1983–2014. *Psychol Women Q.* 2016;40(3):353–63.
3. Hentschel T, Heilman ME, Peus CV. The multiple dimensions of gender stereotypes: A current look at men's and women's characterizations of others and themselves. *Front Psychol.* 2019;10:11.
4. Eagly AH, Nater C, Miller DI, Kaufmann M, Sczesny S. Gender stereotypes have changed: A cross-temporal meta-analysis of U.S. public opinion polls from 1946 to 2018. *Am Psychol.* 2020;75(3):301–15.
5. Prentice DA, Carranza E. What women and men should be, shouldn't be, are allowed to be, and don't have to be: The contents of prescriptive gender stereotypes. *Psychol Women Q.* 2002;26(4):269–81.
6. Rudman LA, Moss-Racusin CA, Phelan JE, Nauts S. Status incongruity and backlash effects: Defending the gender hierarchy motivates prejudice against female leaders. *J Exp Soc Psychol.* 2012;48(1):165–79.
7. Rudman LA, Phelan JE. Backlash effects for disconfirming gender stereotypes in organizations. *Res Organ Behav.* 2008;28:61–79.
8. Heilman ME, Wallen AS. Wimpy and undeserving of respect: Penalties for men's gender-inconsistent success. *J Exp Soc Psychol.* 2010;46(4):664–7.
9. Moss-Racusin CA. Male backlash: Penalties for men who violate gender stereotypes. In: Burke RJ, Major DA, editors. *Gender in organizations: Are men allies or adversaries to women's career advancement?* Edward Elgar Publishing; 2014.
10. Rudman LA. Self-promotion as a risk factor for women: The costs and benefits of counterstereotypical impression management. *J Pers Soc Psychol.* 1998;74(3):629–45.

11. Rudman LA, Glick P. Prescriptive gender stereotypes and backlash toward agentic women. *J Soc Issues.* 2001;57(4):743–62.
12. Phelan JE, Moss-Racusin CA, Rudman LA. Competent yet out in the cold: Shifting criteria for hiring reflect backlash toward agentic women. *Psychol Women Q.* 2008;32(4):406–13.
13. Eagly AH, Makhijani M, Klonsky BG. Gender and the evaluation of leaders: A meta-analysis. *Psychol Bull.* 1992;112(3):557–81.
14. Carli LL. Gender and social influence. *J Soc Issues.* 2001;57(4):725–41.
15. Heilman ME, Wallen AS, Fuchs D, Tamkins MM. Penalties for success: Reactions to women who succeed at male gender-typed tasks. *J Appl Psychol.* 2004;89(3):416–27.
16. Heilman ME, Okimoto TG. Why are women penalized for success at male tasks?: The implied communality deficit. *J Appl Psychol.* 2007;92(1):81–92.
17. Moss-Racusin CA, Phelan JE, Rudman LA. When men break the gender rules: Status incongruity and backlash against modest men. *Psychol Men Masc.* 2010;11(2):140–51.
18. Raymondie RA, Steiner DD. Backlash against counter-stereotypical leader emotions and the role of follower affect in leader evaluations. *J Appl Soc Psychol.* 2022;52(8):676–92.
19. Rosette AS, Mueller JS, Lebel RD. Are male leaders penalized for seeking help? The influence of gender and asking behaviors on competence perceptions. *Leadersh Q.* 2015;26(5):749–62.
20. Rudman LA, Mescher K. Penalizing men who request family leave: Is flexibility stigma a femininity stigma? *J Soc Issues.* 2013;69(2):322–40.
21. Moss-Racusin CA, Johnson ER. Backlash against male elementary educators. *J Appl Soc Psychol.* 2016;46(5):379–93.
22. Toneva Y, Heilman ME, Pierre G. Choice or circumstance: When are women penalized for their success? *J Appl Soc Psychol.* 2020;50(11):651–59.
23. Heilman ME, Martell RF, Simon MC. The vagaries of sex bias: Conditions regulating the undervaluation, equivaluation, and overvaluation of female job applicants. *Organ Behav Hum Decis Process.* 1988;41(1):98–110.

24. Lanaj K, Hollenbeck JR. Leadership over-emergence in self-managing teams: The role of gender and countervailing biases. *Acad Manag J.* 2015;58(5):1476–94.
25. Schaumberg RL, Flynn FJ. Self-reliance: A gender perspective on its relationship to communality and leadership evaluations. *Acad Manag J.* 2017;60(5):1859–81.
26. Ma A, Rosette AS, Koval CZ. Reconciling female agentic advantage and disadvantage with the CADDIS measure of agency. *J Appl Psychol.* 2022;107(12):2115–48.
27. Rudman LA, Glick P. Feminized management and backlash toward agentic women: The hidden costs to women of a kinder, gentler image of middle managers. *J Pers Soc Psychol.* 1999;77(5):1004–10.
28. Heilman ME, Chen JJ. Same behavior, different consequences: Reactions to men's and women's altruistic citizenship behavior. *J Appl Psychol.* 2005;90(3):431–41.
29. Hentschel T, Braun S, Peus C, Frey D. The communality-bonus effect for male transformational leaders–leadership style, gender, and promotability. *Eur J Work Organ Psychol.* 2018;27(1):112–25.
30. Krstic A, Hideg I. The effect of taking a paternity leave on men's career outcomes: The role of communality perceptions. *Acad Manag Proc.* 2019;2019(1):278.
31. Heilman ME. Description and prescription: How gender stereotypes prevent women's ascent up the organizational ladder. *J Soc Issues.* 2001;57(4):657–74.
32. Heilman ME. Gender stereotypes and workplace bias. *Res Organ Behav.* 2012;32:113–35.
33. Eagly AH, Karau SJ. Role congruity theory of prejudice toward female leaders. *Psychol Rev.* 2002;109(3):573–98.
34. Eagly AH, Wood W. Social role theory of sex differences. In: Naples N, Hoogland R, Wickramasinghe C, et al., editors. *The Wiley Blackwell encyclopedia of gender and sexuality studies.* Wiley Blackwell; 2016.
35. Jussim L, Coleman LM, Lerch L. The nature of stereotypes: A comparison and integration of three theories. *J Pers Soc Psychol.* 1987;52(3):536–46.

36. Eareckson HB, Heilman ME. Explaining penalties and rewards for gender norm violations: A unified theory. *Sex Roles.* 2024;90:1701–16.
37. Koenig AM. Comparing prescriptive and descriptive gender stereotypes about children, adults, and the elderly. *Frontiers in psychology*. 2018 Jun 26;9:1086.
38. Ghavami N, Peplau LA. An intersectional analysis of gender and ethnic stereotypes: Testing three hypotheses. *Psychol Women Q.* 2013;37(1):113–27.
39. Sanchez-Hucles JV, Davis DD. Women and women of color in leadership: Complexity, identity, and intersectionality. *Am Psychol.* 2010;65(3):171–81.
40. Rosette AS, de Leon RP, Koval CZ, Harrison DA. Intersectionality: Connecting experiences of gender with race at work. *Res Organ Behav.* 2018;38:1–22.
41. Hall EV, Galinsky AD, Phillips KW. Gender profiling: A gendered race perspective on person–position fit. *Pers Soc Psychol Bull.* 2015;41(6):853–68.
42. Zarate MA, Smith ER. Person categorization and stereotyping. *Soc Cogn.* 1990;8(2):161–85.
43. Cheryan S, Markus HR. Masculine defaults: Identifying and mitigating hidden cultural biases. *Psychol Rev.* 2020;127(6):1022–52.
44. Williams J. *Unbending gender: Why family and work conflict and what to do about it.* Oxford University Press; 2001.
45. Galinsky AD, Hall EV, Cuddy AJ. Gendered races: Implications for interracial marriage, leadership selection, and athletic participation. *Psychol Sci*. 2013 Apr;24(4):498-506.
46. Johnson KL, Freeman JB, Pauker K. Race is gendered: How covarying phenotypes and stereotypes bias sex categorization. *J Pers Soc Psychol.* 2012 Jan;102(1):116-31.
47. Livingston RW, Rosette AS, Washington EF. Can an agentic Black woman get ahead? The impact of race and interpersonal dominance on perceptions of female leaders. *Psychol Sci.* 2012 Apr;23(4):354-8.

Click here to access/download;Figure;Fig. 1.png

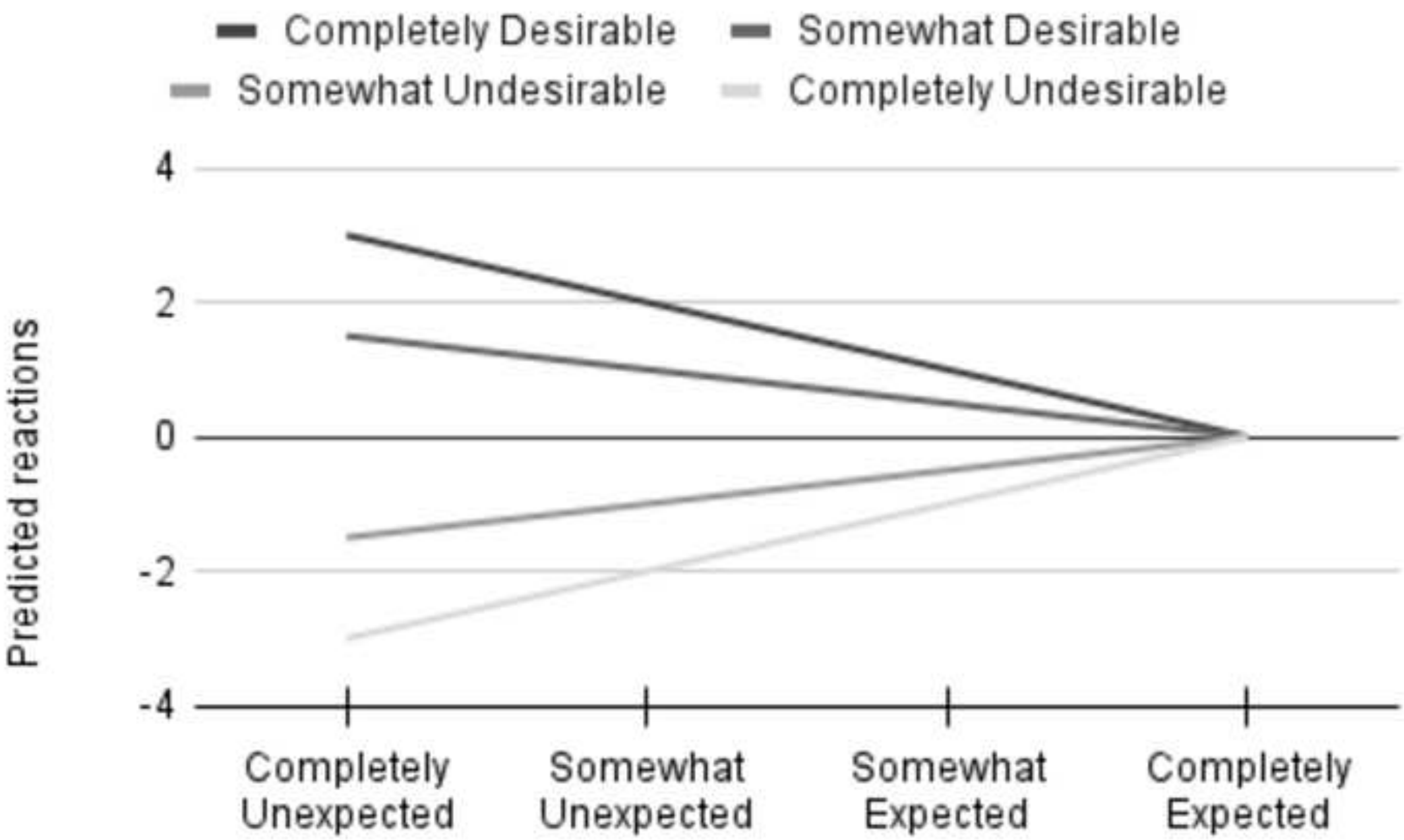

Figure

Click here to access/download;Figure;Fig. 2.png

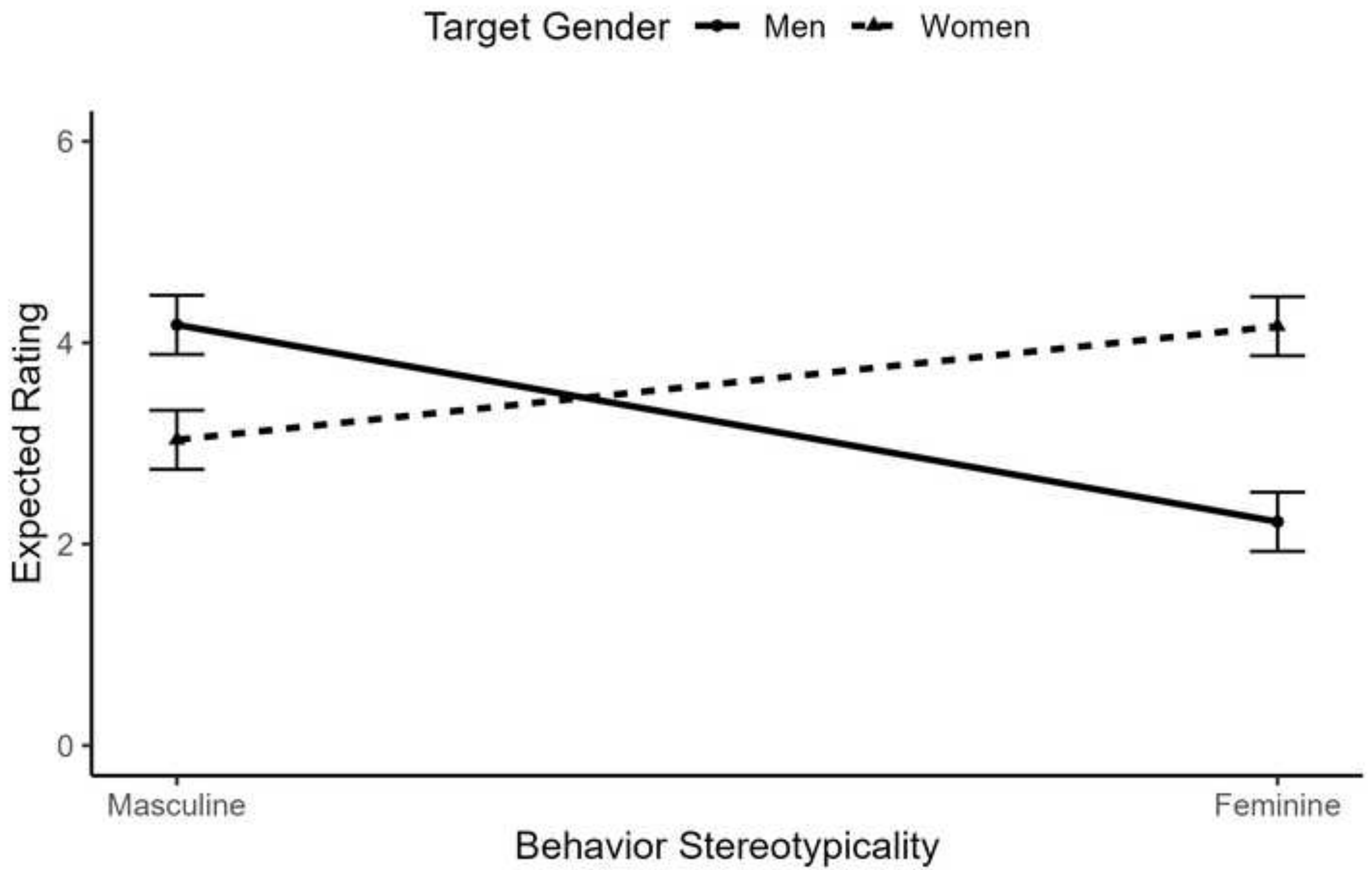

Figure

Click here to access/download;Figure;Fig. 3.png

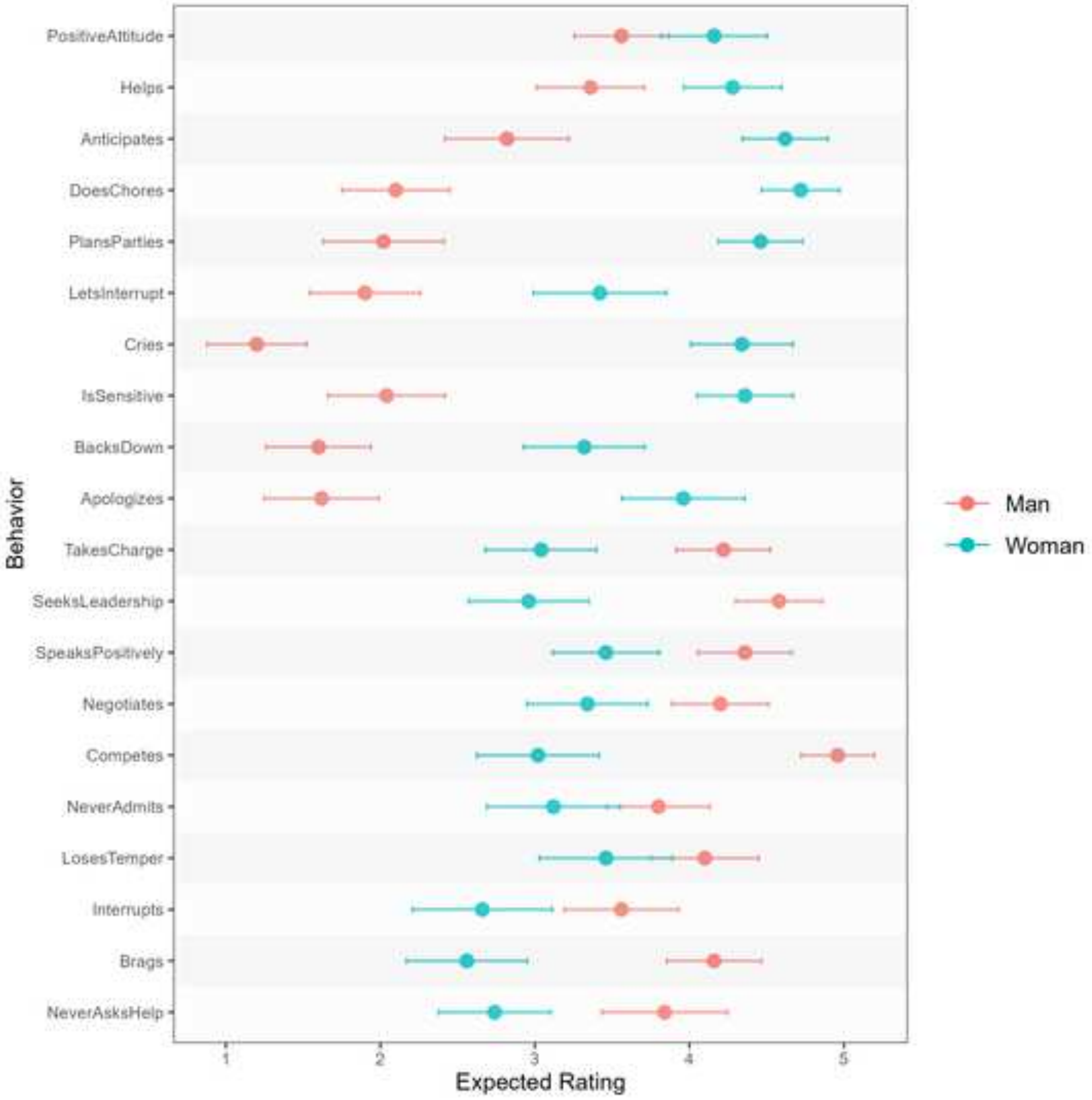

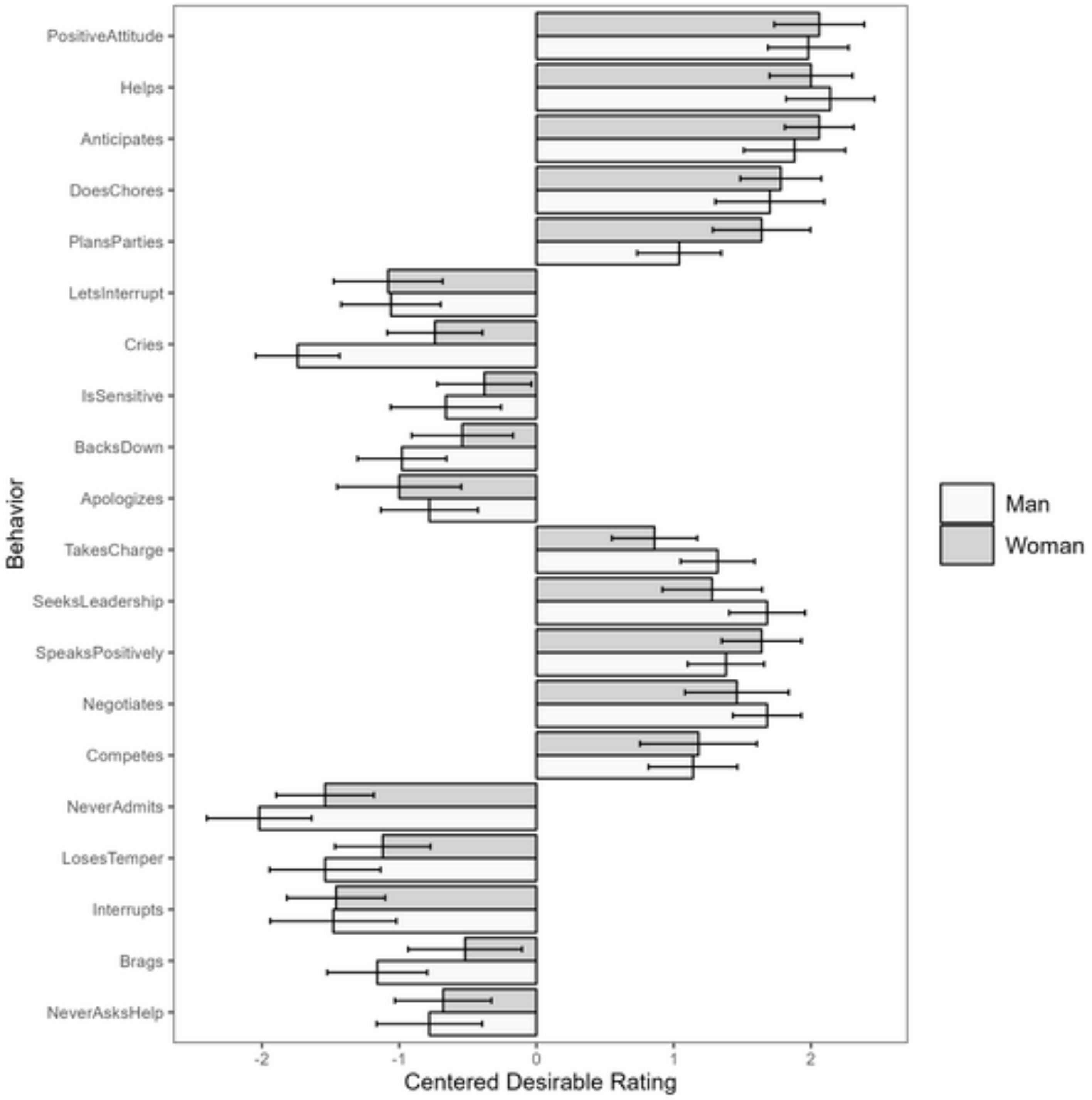
PositiveAttitude
Helps
Anticipates
DoesChores
PlansParties
LetsInterrupt
Cries
IsSensitive
BacksDown
Apologizes
TakesCharge
SeeksLeadership
SpeaksPositively
Negotiates
Competes
NeverAdmits
LosesTemper
Interrupts
Brags
NeverAsksHelp
Behavior
-2
-1
0
1
2
Centered Desirable Rating
Man
Woman

 Click here to access/download;Figure;Fig. 5.png

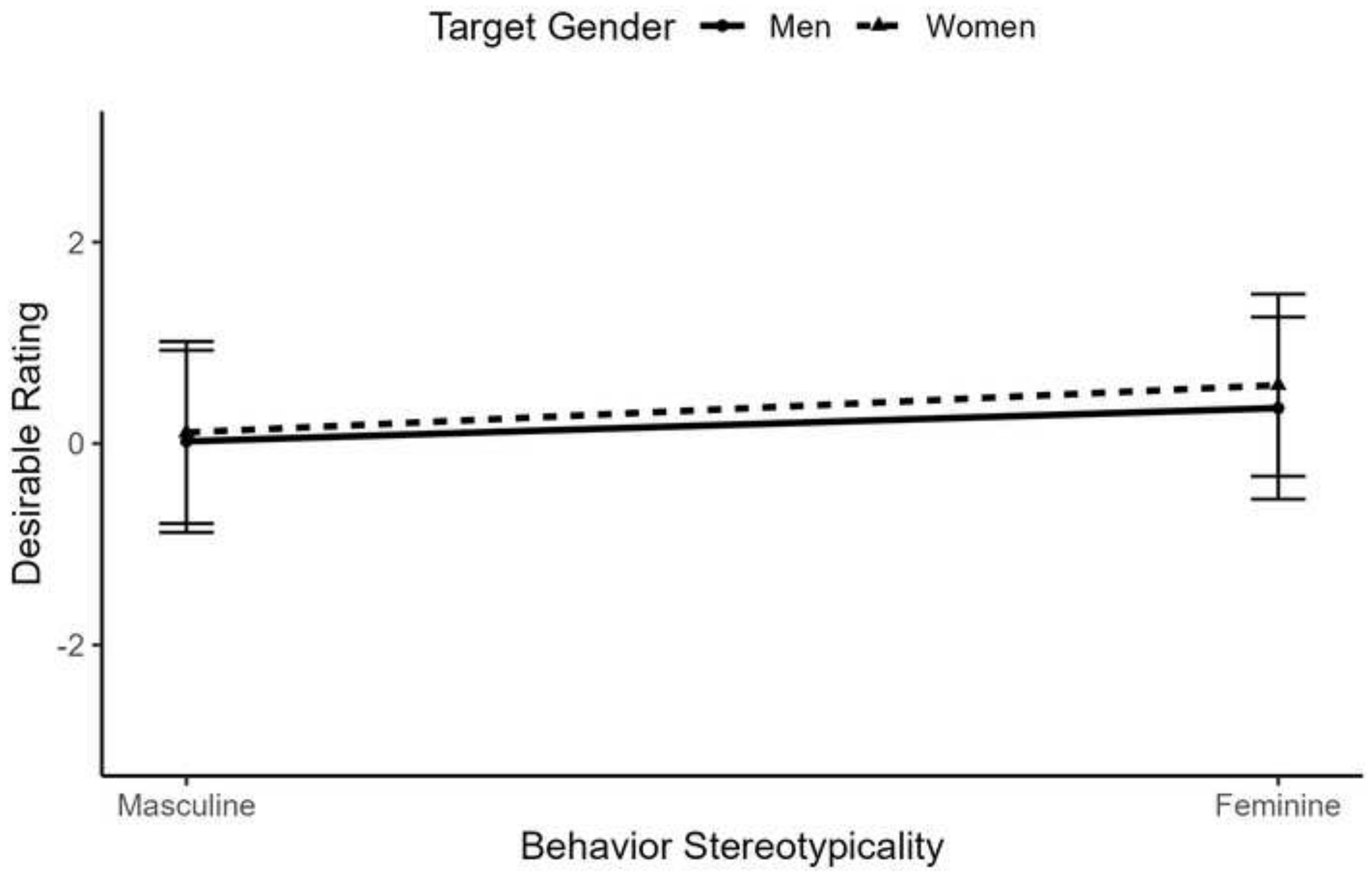

Figure

Click here to access/download;Figure;Fig. 6.png

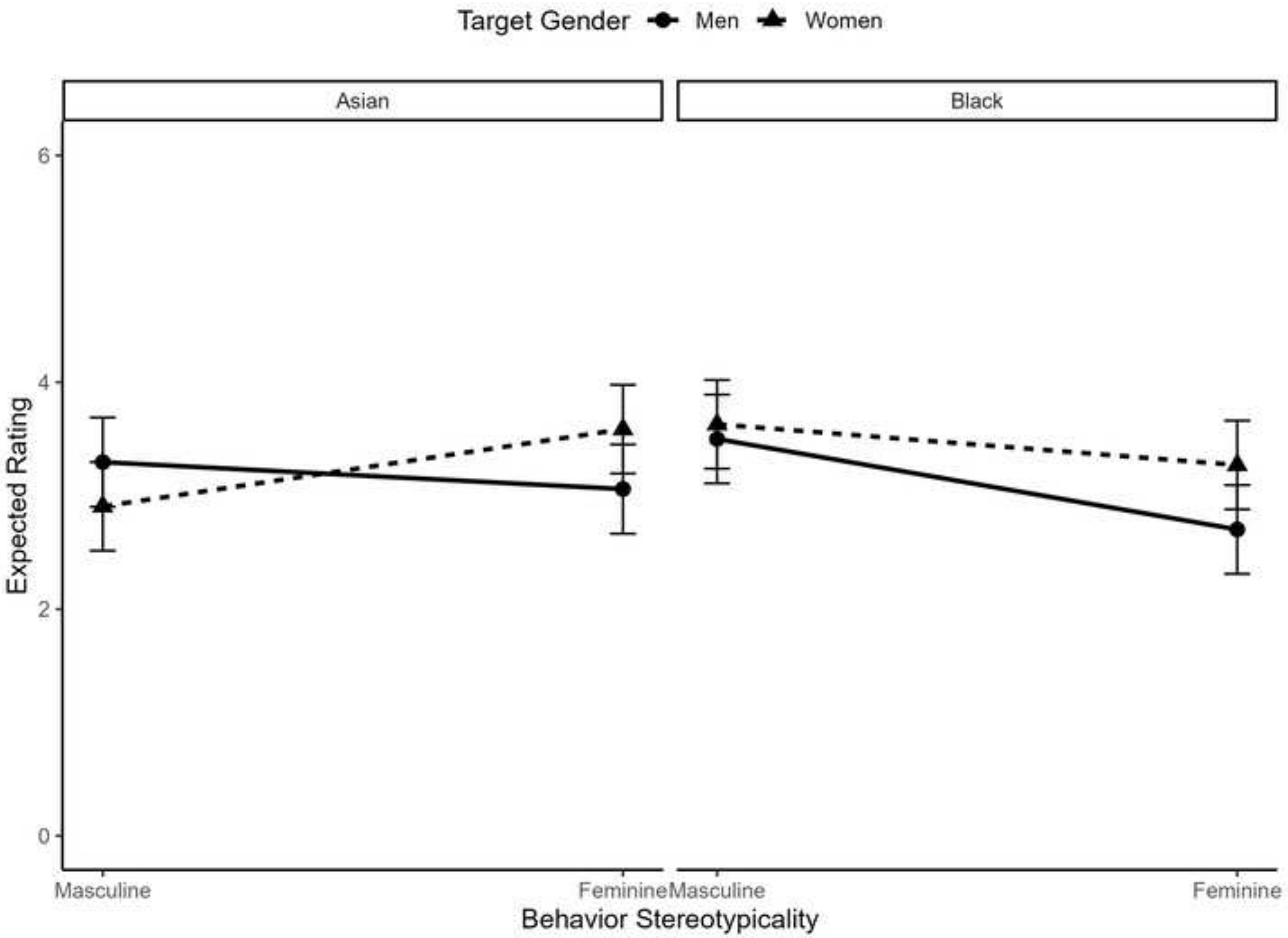

Figure Click here to access/download;Figure;Fig. 7.png

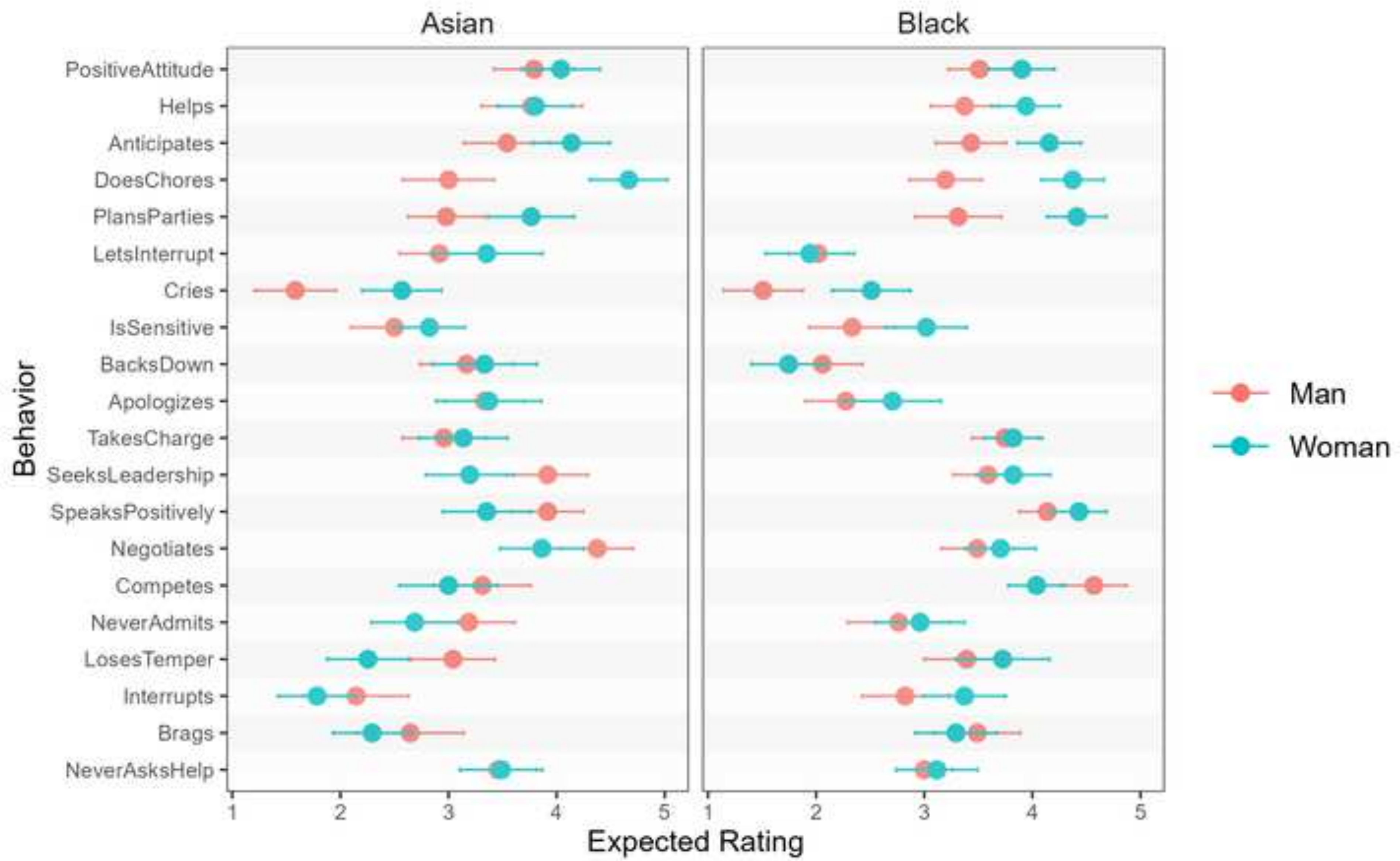

Figure

Click here to access/download;Figure;Fig. 8.png

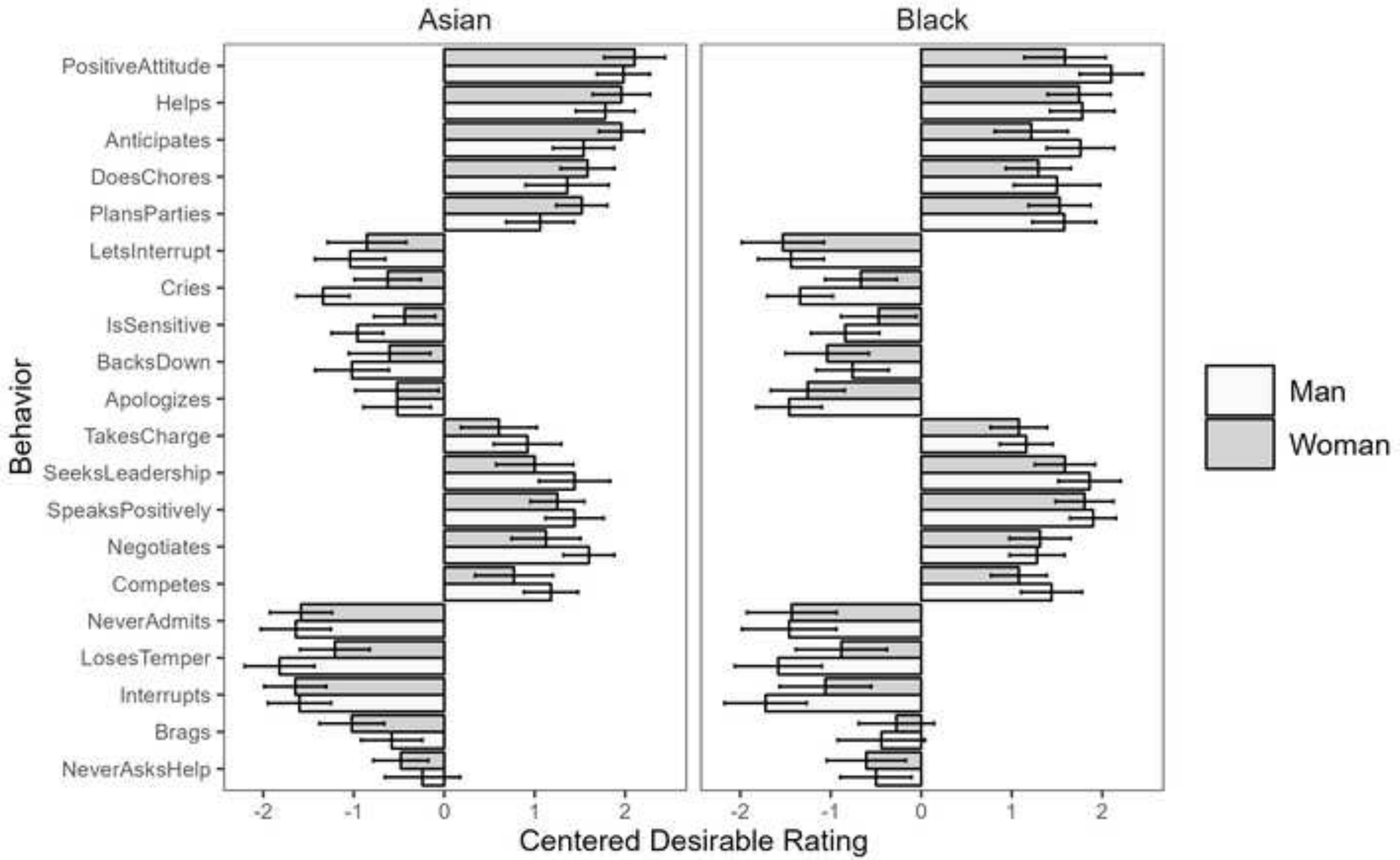

Figure

Click here to access/download;Figure;Fig. 9.png

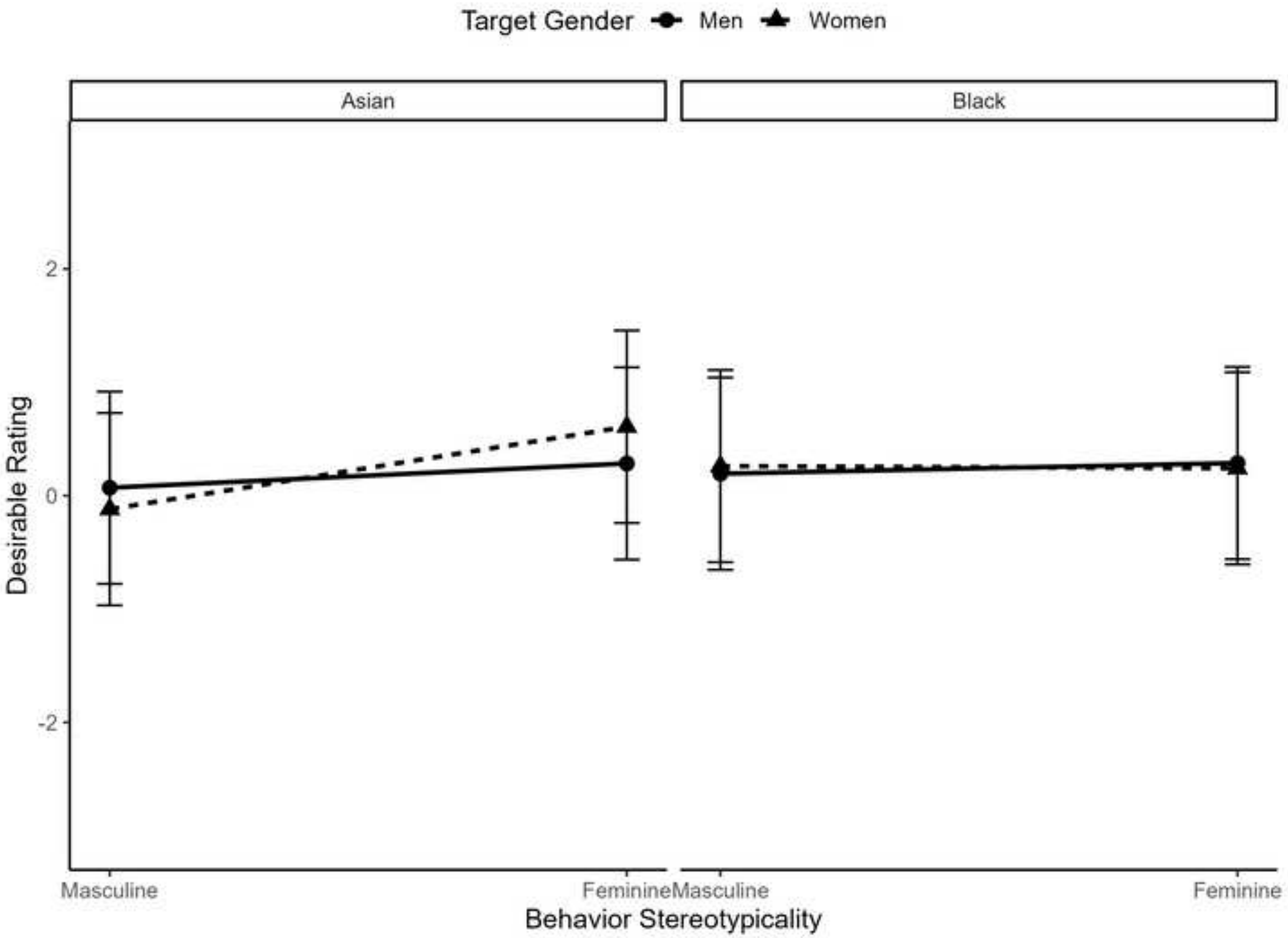

Figure

Click here to access/download;Figure;Fig. 10.png

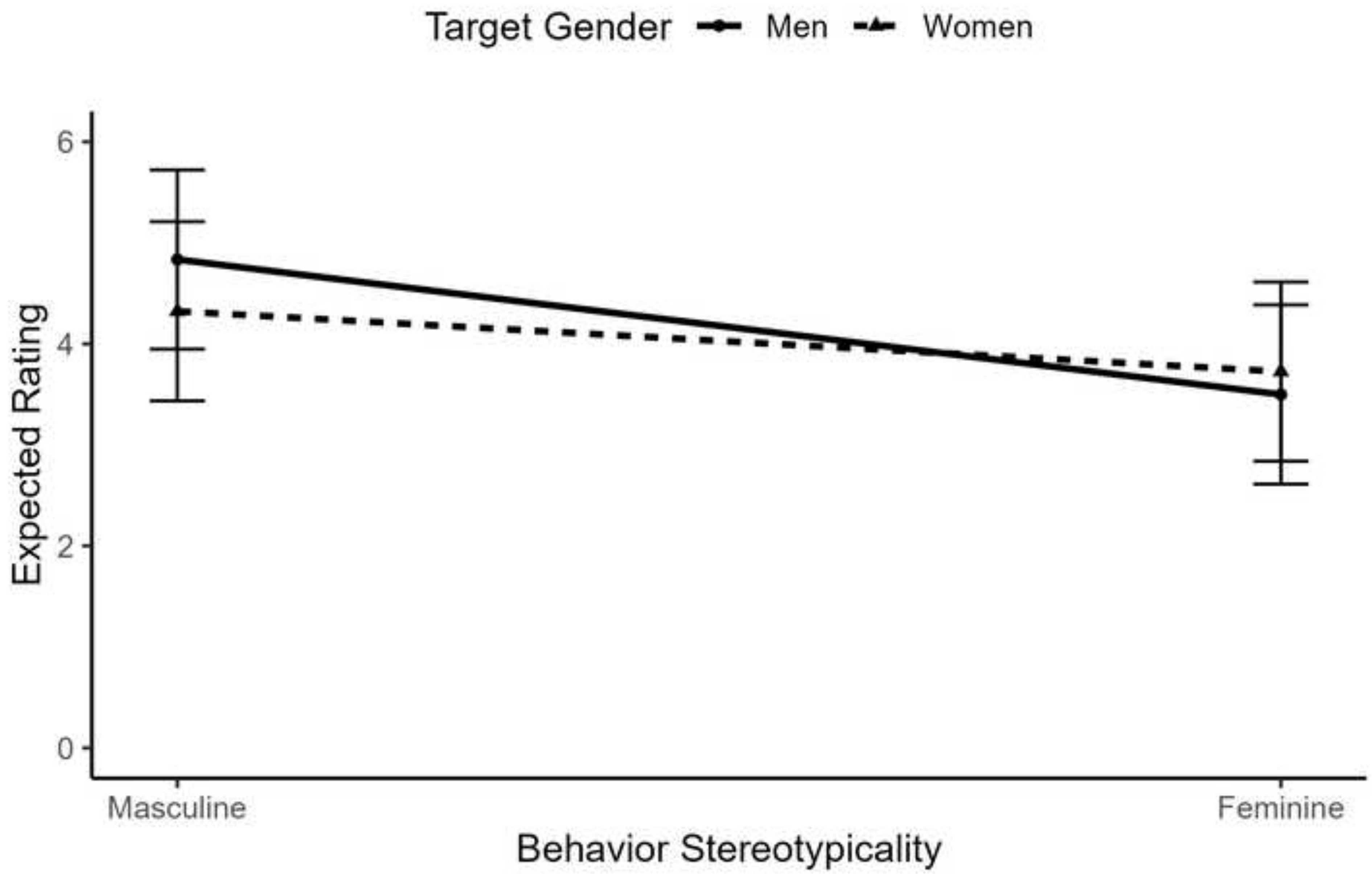

Figure

Click here to access/download;Figure;Fig. 11.png

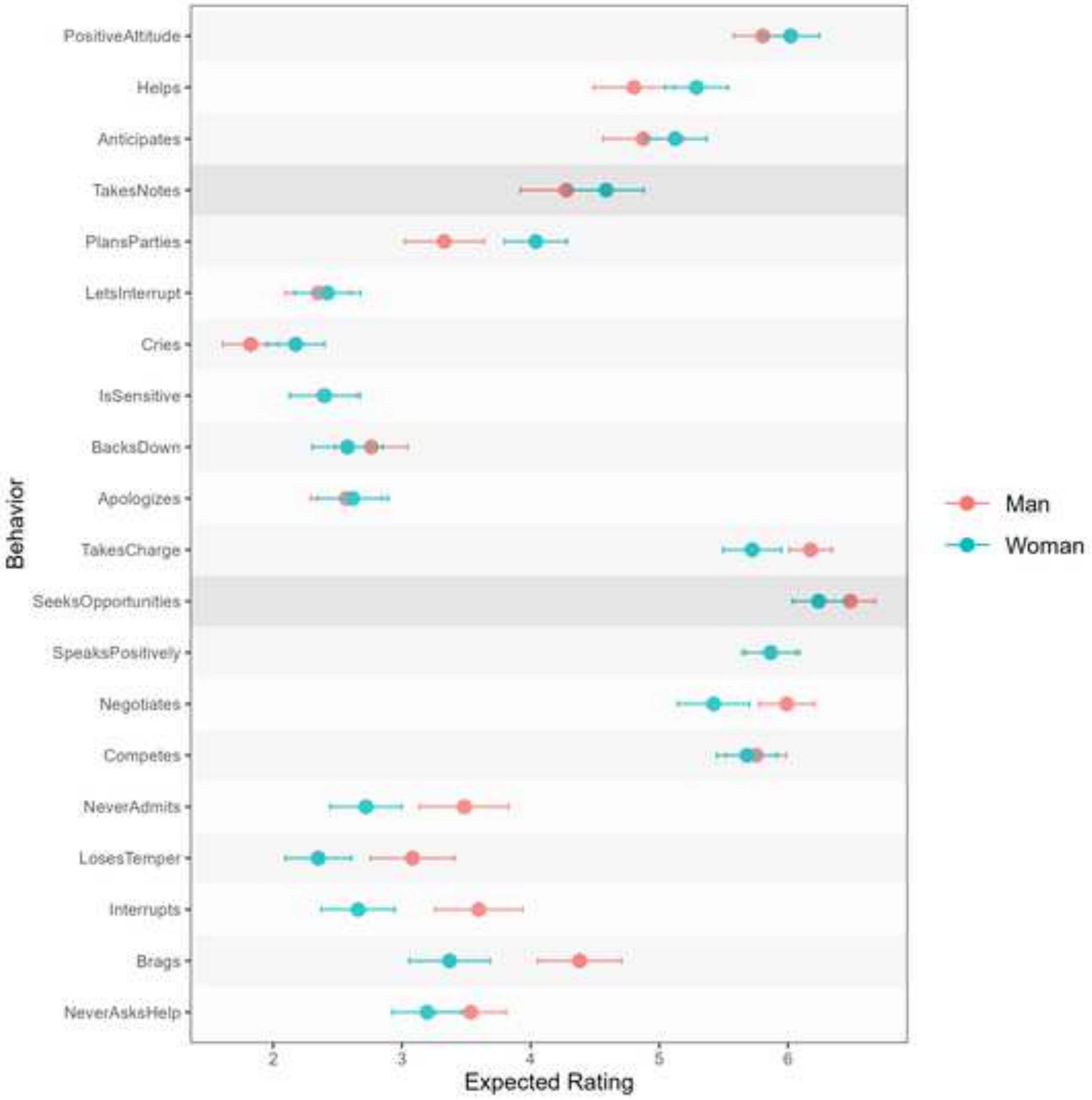

Click here to access/download;Figure;Fig. 12.png

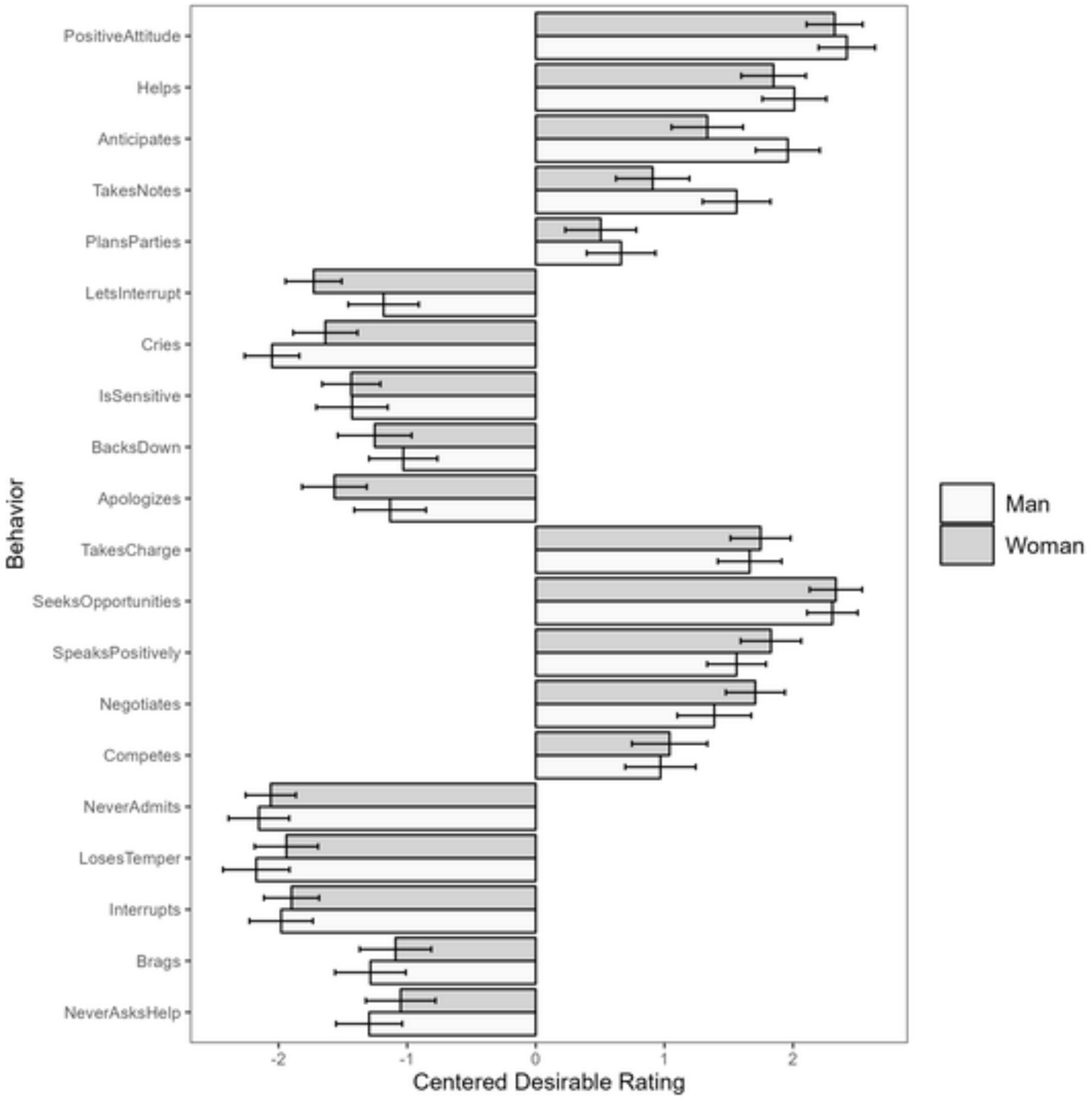

Figure

Click here to access/download;Figure;Fig. 13.png

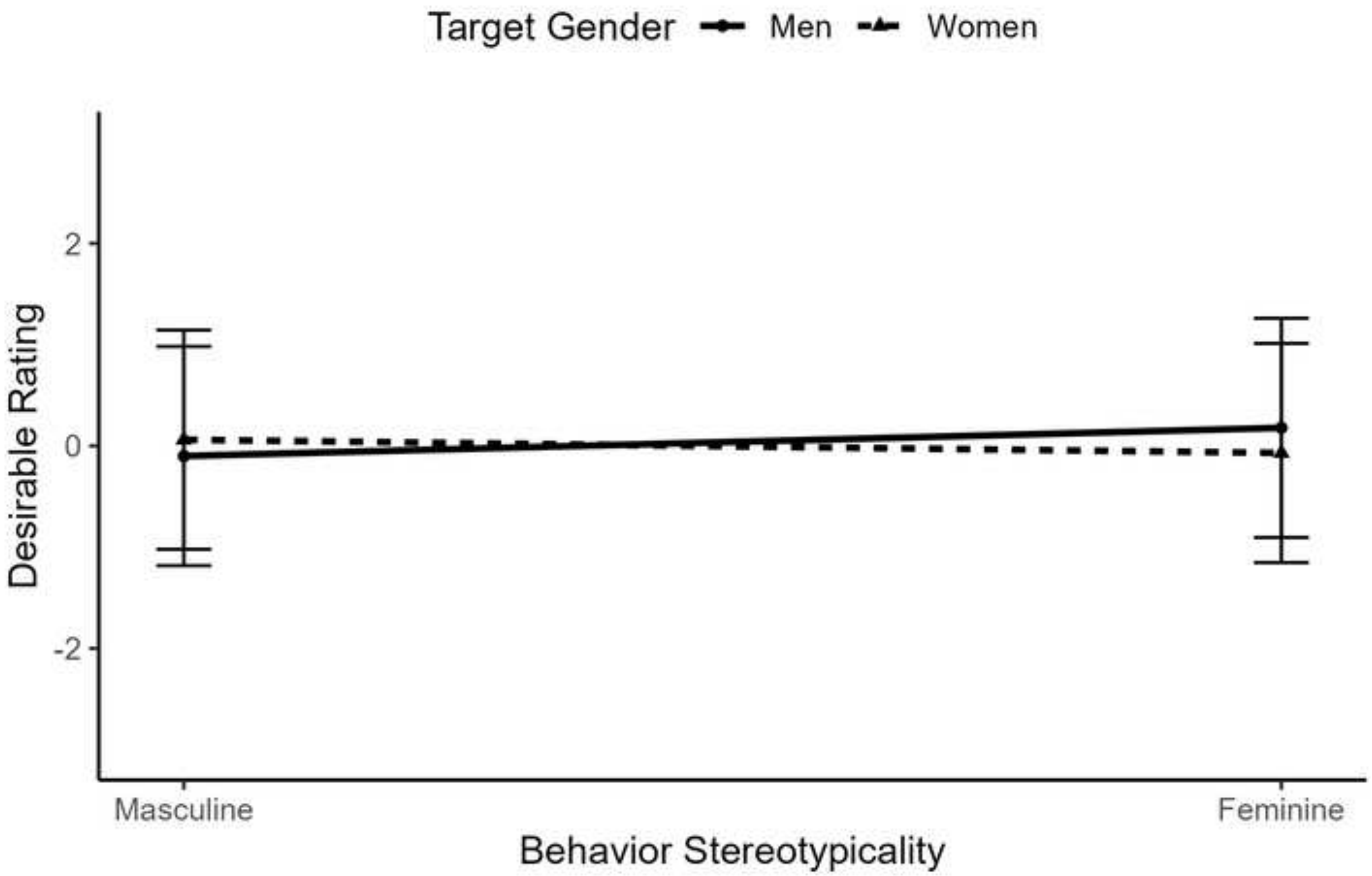

Supplement: Attachment — Submitted filename: PONE-D-25-13132.pdf [file pone.0343921.s002.pdf]
