# Supplementary material for: Is what’s unexpected undesirable? Investigating the expectedness and desirability of gender-stereotypic behaviors in men and women
Source: PLoS One. 2026 Apr 1;21(4):e0343921. doi: 10.1371/journal.pone.0343921 (PMC13042864; doi:10.1371/journal.pone.0343921)
Supplement: Attachment — Submitted filename: PLOS ONE_Revision Letter (1).docx [file pone.0343921.s004.docx]

Dear Dr. Mazrekaj,

Thank you so much for the great feedback from yourself and the reviewers, as well as the opportunity to revise and resubmit our manuscript. We greatly appreciate the feedback that both you and the reviewers have provided; we have addressed all of the comments and feel that the paper is much improved in terms of its theoretical contribution, its clarity, and the thoroughness of our discussion of our methodological choices, differences according to participant identity, and the limitations of the designs we used. Below, we provide our specific changes and responses to each comment. We believe that the current manuscript addresses all of the reviewer comments and is far improved for it, but we would be more than happy to further edit the manuscript if you or any of the reviewers feel that there are further changes which could improve our contribution.

**Editor comments:**

*- What is the substantive and theoretical contribution of the study? It appears that this theoretical framework was already proposed by the authors in another paper.*

- Thank you for suggesting we clarify the contribution; we feel that our revisions to the introduction have substantially enhanced the contribution of the paper. In the introduction, we further explain how this paper expands on and supports the theoretical framework we proposed in Eareckson and Heilman (2024) - on pgs. 5-6, we expanded on the theoretical limitations of the traits used by Eareckson & Heilman (2024) to examine these theories, and made clearer the conceptual value of directly testing the assumptions of role congruity theories, expectancy violation theory, and the proposed unified theory through an examination of the expectedness and desirability of gender-typical behaviors, as we do here. In addition, we feel that our explanation of our decision to directly ask participants about their own perceptions (rather than asking about perceptions of views in society, as past work as done to get around desirability affects), makes more clear the contribution of these studies.

*- There is too little methodological information (sample and methods) to understand the results. This part should be thoroughly revised paying particular attention on whether the sample is representative and who the participants are exactly.*

- Thank you for this advice; we have entirely revised our description of our methods and sample by condensing this information to a single section (p. 8) for the full paper; we moved the description of the behavior pilot-testing earlier in the paper, and separated the specific question wording and participant demographics in each study into a table (p. 10), as suggested by Reviewer 1, to make clear the methodological differences across studies, and the wording used in each dependent variable measure. Additionally, this allows for clear comparison of sample demographics across studies; we also added tests of moderating effects of participant gender and race identity throughout the manuscript, as suggested by all three reviewers.

*- The manuscript is currently too long and often repetitive. It needs to be shortened.*

- We have substantially streamlined and simplified the presentation of the study results to shorten the paper and prevent repetition. Although the expansion of the theoretical contributions, limitations, and the added moderation analyses requested by reviewers have counter-acted our efforts to shorten the paper in terms of length, we feel that these additions substantially enhance the contribution of the paper, and that the simplification of the study presentations has resulted in a much cleaner and less repetitive paper.

**Review 1 Comments**

*The authors provide detailed information on the design and participant composition for each of the six studies in the texts. Since the studies are complementary and com-parable, I suggest creating a summary table to present this more clearly and succinctly. For example, rows could include: “Question Asked”, “Hypothesis tested”, “Number and Share of Female Participants”, “Number and Share of White Participants”, “Total Number of Participants”, and “Average Age of Participants”, with columns for each of the six studies. This would provide a more straightforward overview of sample sizes and demographics, and allow the main text to focus on differences across studies. For instance, in Study 2a and 2b, the gender imbalance is larger than in the other studies.*

- Thank you very much for this wonderful suggestion! We feel that this greatly improves the clarity of the designs and samples across studies, and helps shorten the body of the manuscript and reduce the repetitiveness of repeating these in each study. We have added a table with this information at the end of the joint methods section (p. 10, Table 3), although we used a different table organization than you proposed to improve the table readability.

*• As acknowledged in the "Limitations" section, participants are primarily White and the samples are not nationally representative. While it is good to be transparent about this issue, I wonder whether and how this limitation might affect the results. One approach to address this would be to replicate the studies with nationally representative samples; however, obtaining such samples is not always feasible. Alternatively, some adjustments or controls might be applied to account for sample composition. For example, they “fit linear mixed models” to test hypotheses 1 and 3, which includes “random intercepts for participant ID and specific behavior”. My understanding is that they (1) use random effects for participant ID to account for repeated measures within each person as each participant rated multiple behaviors; and (2) use random effects for specific behavior to account for the differences in the average levels of expectedness or desirability across behaviors. It would be helpful if the authors could briefly discuss whether any adjustments for sample composition are feasible within this modeling framework, and or what types and directions of bias might arise.*

- Thank you for recommending this; we have indeed found some interesting moderations due to participant gender, race, age, and political orientation, which are now reported in the main text of the paper (pgs. 12, 13, 15, 16, 17), and summarized in the discussion (p. 19). We have compiled these moderation tests at the end of the letter and highlighted the significant moderations in each study, for ease of reference.

*• A minor point: in Figures 3, 7, and 11, it is difficult to distinguish results by gender when printed in black and white. I suggest using different symbols (e.g., circles for men and triangles for women) to improve clarity.*

- Thank you again; we have taken your advice, and the figures have been replaced with circles and triangles to improve clarity!

**Reviewer #2 Comments**

*The study is quite interesting as it seeks to understand how gender-typicality affects how much people expect and desire certain gender-stereotypic behaviors. However, I have concerns with the demographics of the respondents especially for study 2 where there are questions asked about the expectations of blacks and asians. With a larger sample of white population, will it be safe to draw conclusions about the traits of blacks or asians when non-blacks and non-asians are the ones being sampled. Or how do we interpret the result of the study, expectations from non-blacks or non-asians about the behaviors of blacks or asians?*

- Thank you for pointing this out! We have acknowledged the limitation of our sampling demographics further in the discussion, and reported tests of the moderations of participant gender, race, age, and political orientation on each of our key interactions (pgs. 12, 13, 15, 16, 17). We have compiled these moderation tests at the end of the letter and highlighted the significant moderations in each study, for ease of reference.
  - For the race moderation, specifically, we do observe differences in how White, Black, and Asian participants respond to the behavior expectedness in Study 2a, but we observe consistent patterns of desirability ratings across race identity in Study 2b. In Study 2a, participant race moderated the three-way interaction between participant race, participant gender, and behavior stereotypicality on expectedness, such that White participants showed gender stereotyping in expectedness for both Asian and Black targets, while Non-White participants showed gender stereotyping for Asian, but not Black, targets. Breaking this down for Asian and Black participants, we found that this effect seemed to be particularly driven by Asian participants, who showed no evidence of gender stereotyping for Black targets. We feel that including these analyses in the paper greatly enhances the interpretation of these studies, allowing for insight into how White, Asian, and Black participants responded differently (or did not respond differently) in these studies.

**Reviewer 3 Comments**

*1) While the authors provide demographic particulars of their participants pool, can they also use this variation to understand better who is more likely to penalize less for gender atypical behaviour? For example, are women less likely to punish women for counter gender-typical behaviour? Certain behaviour like crying seems to be clearly less desirable for men, as the authors note. Maybe pooling all studies and examining differences by men vs women participants or by participant age or electoral preferences, can throw some light on whether some demographic groups rate behaviour differently*

- Thank you for this great suggestion for looking at the data! In line with the recommendations of Curran and Hussan’s (2009) IDA technique to test for pooled effects, we combined our data and created effects-coded variables to represent study number (in this case, variable 1 comparing Studies 1 (coded -.5) and 3 (coded .5), excluding Study 2 (coded 0), and variable 2 comparing Study 2 (coded 1) to the others (coded -.5 for both)). Since we saw numerous interactions with these study variables across our tests, and since this technique is complex, was not pre-registered in our analysis plan, and would require an additional section at the end of the paper explaining the technique and all the results (only to go back and break down these results by study, due to the significant interactions), we chose to conduct separate moderation analyses for participant gender, race, age, and political orientation; we now report these results for the main interaction (target gender * behavioral stereotypicality) across all studies in the main text. We include a summary of all moderating interactions at the end of this letter, so that you can review these findings and see if you agree with our assessment.
  - In addition, we try to avoid over-interpreting these results in our paper, as they were exploratory (and many), and thus may result in an inflated Type I error rate due to the increased number of tests run. We summarize them in our discussion, and conclude with this suggestion for future research: “Since these tests of moderation were only exploratory, and not consistent across target identities, we hesitate to place too much emphasis on interpreting any of these effects; however, we hope that these interactions can provide insight for researchers looking to expand on how and why individuals with varying backgrounds and identities may apply different gender-based stereotypes and norms.” (pg. 19)

*2) Demographic composition is not similar across studies – men range from 35% to 50% - it was not clear in the paper but did the authors control for participant demographics when examining differences in expected behaviour and desirable behaviour?*

- We did not adjust for demographic differences in our key analyses, but we have added tests of the moderation of participant gender, race, age, and political orientation on each of our key analyses, which we feel address these concerns more thoroughly than including them as statistical adjustments (which would not provide insight into whether or not these evaluations *differ* according to demographic identity). (pgs. 12, 13, 15, 16, 17)

*3) Did the authors take any steps to minimize social desirability in reporting in survey by participants? If the authors captured any social desirability index, they should control for it in their analyses or recognize the possible role that it can play in the obtained results.*

- Unfortunately, we did not capture a social desirability index, so we cannot adjust for that in our analyses. We have now added a paragraph explaining our choice to ask participants their personal views, rather than asking them to estimate others’ views, as research on stereotypes and norms often does (p. 6).
  - “Second, many investigations of gender stereotypes and norms aim to avoid social desirability effects by asking participants to evaluate how typical or desirable others in society would view a particular trait in men and women, rather than directly asking participants to provide their own evaluations of stereotypicality or desirability (i.e., Prentice and Carranza (2002) asked participants “how desirable it is *in American society* for a man to possess each of these characteristics”). This is very useful for assessing the perception of societal norms (i.e., how do men and women *think* these behaviors are viewed in society), but not as useful for assessing individuals’ own views on these behaviors.”
- We acknowledge that we cannot completely eliminate this possibility, but that an anonymous online survey format should hopefully mitigate these characteristics. (p. 6)
  - “That is what we do in the current studies, using an online survey format to mitigate social desirability effects as much as possible. Although the motivation to appear unbiased may taint participants’ responses… asking participants their direct opinions is the best way to ascertain participants’ true views, even if it is imperfect.”

*4) The statement used by authors are very generic – “How expected do you think this behavior is for a TYPICAL [MAN/WOMAN]?” on a seven-point scale ranging from ‘completely unexpected’ (0) to ‘completely expected’ (6). “ Similarly, they ask about desirability. While in general, a person can be okay with women in leadership but may not be desirable if one were to have a mother or a partner in such a role since it is not congruent with being typical of women. The authors should caveat their findings more carefully—because in a personal circumstance a trait may not be desirable even though in general it does not matter.*

- We very strongly agree with this statement, and we feel that our diverging results for typical, Black and Asian, and successful working women are actually in support of this assertion. Our observation that work context does change effects supports this conclusion, and future work should focus on the specific context they are studying the behavior in. We were interested here in work environments, but it is likely that specific contexts will have very different effects on perceptions of behavioral expectedness and desirability - we expand on this point in the conclusion (p. 19) when we discuss the implications of our findings for contextual differences in these evaluations, and note the domain of romantic relationships as a context of particular interest for gender differences in behavioral perceptions.
- In the limitations, we also discuss further how these broad construals of behaviors may not reflect individuals’ perceptions in more individuated interpersonal contexts:
  - “Therefore, our findings can only truly speak to how individuals think they would feel about a general man or woman acting in the described ways; in personal interactions, with more nuanced and specific contextual information and personal histories, these perceptions will likely differ from the broad construals we study. We chose to examine high-level perceptions of gender-stereotypic behaviors by asking participants to provide their impressions of behaviors with a one-line description. In reality, these behaviors take place in complex social settings and can be enacted in a variety of ways; future researchers can build on our findings by studying perceptions of gender-typical behaviors in more specific and individual interaction contexts.” (p. 20)

Minor comments:

*1) The authors can consider providing what was the exact question asked in each study to the participants in the text of the paper.*

- Thank you for this suggestion! We have incorporated Reviewer 1’s suggestion of a summary table including the specific question text, hypotheses, and participant demographics in each of the six studies; we believe this provides valuable clarification of the exact methods and questions asked in these studies. (p. 10)

Again, we thank you for the opportunity to revise and resubmit our paper for publication in PLOS ONE. We look forward to hearing your thoughts on this revised version of the manuscript, and believe that publishing open access in PLOS ONE is the best outlet for our work to maximize its impact and reach a large, cross-disciplinary audience.

Sincerely,

Hannah Eareckson and Madeline Heilman

**Compiled identity moderation effects - significant moderations are highlighted; these are all reported in the main text of the manuscript, as well.**

Study 1a: Relative expectedness differences for typical targets (included)

- Participant gender moderated this interaction, *b* = .20, *t*(1878) = 6.65, *p* < .001, such that the interaction was significant and consistent in direction for both male and female participants, but stronger for female participants, *b* = .94, *t*(1082) = 24.85, *p* < .001, than for male participants, *b* = .55, *t*(740) = 11.98, *p* < .001. Participant race also moderated this interaction, *b* = .30, *t*(1878) = 4.54, *p* < .001, such that the interaction was significant and consistent in direction for both White and Non-White participants, but stronger for White participants, *b* = .86, *t*(1329) = 24.99, than for Non-White participants, *b* = .57, *t*(531) = 10.22, *p* < .001. Participant political orientation also moderated the interaction between target gender and behavioral stereotypicality, b = .09, t(1830) = 4.74, p < .001, such that it was stronger for liberal participants than conservative participants. Participant age was not a significant moderator, b < .01, t(1840) = .60, p = .549.

Study 1b: Relative desirability differences for typical targets (included)

- Participant gender did not moderate this interaction, *b* = -.24, *t*(1878) = .84, p = .404, nor did participant race, *b* < -.01, *t*(1878) = .03, *p* = .974, nor age, *b* < .01, *t*(1859) = 1.76, *p* = .079. Participant political orientation moderated the interaction between target gender and behavior stereotypicality, *b* = -.04, *t*(1859) = 2.64, *p* = .008, such that more conservative participants showed the predicted interaction, rating feminine behaviors as relatively more desirable in women than in men, and masculine behaviors as relatively more desirable in men than women, but more liberal participants did not.

Study 2a: Relative expectedness differences for typical Black and Asian targets

- Interaction between target gender and behavior stereotypicality (included)
  - Participant gender did not moderate the two-way interaction between target gender and behavior stereotypicality, *b* = .03, *t*(3793) = 1.27, *p* = .204, nor did participant race, *b* = .01, *t*(3793) = .21, *p* = .836, age, *b* < .01, *t*(3774) = 1.08, *p* = .282, or political orientation, *b* = -.01, *t*(3774) = .80, *p* = .424.
- Interaction between target race and behavior stereotypicality (not included)
  - Participant gender did not moderate the two-way interaction between target race and behavior gender-typicality, *b* < -.01, *t*(3793) = .21, *p* = .837, nor did participant race, *b* = .07, *t*(3755) = 1.50, *p* = .134, or age, *b* < .01, *t*(3774) = .07, *p* = .942. The interaction was moderated by political orientation, *b* = .04, *t*(3774) = 3.18, *p* = .002, such that it was stronger for conservative participants than for liberal participants.
- Three way interaction
  - The three-way interaction was not moderated by participant gender, *b* = -.03, *t*(3793) = 1.47, *p* = .142, age, *b* < .01, *t*(3774) = 1.14, *p* = .252, or political orientation, *b* = -.01, t(3774) = .75, *p* = .453. However, participant race did moderate the three-way interaction, *b* = .10, *t*(3793) = 2.24, *p* = .024. White participants followed the general patterns observed in the sample, while non-White participants applied gendered behavioral expectations to Asian targets, *b* = .28, *t*(892) = 6.15, *p* < .001, but not to Black targets, *b* = .06, *t*(968) = 1.45, *p* = .146. Further breaking down this effect by participant race, we found that Black participants applied stereotypes to Asian targets, *b* = .30, *t*(360) = 4.41, *p* < .001, but only marginally to Black targets, *b* = .13, *t*(379) = 1.90, *p* = .058, and that Asian participants also applied stereotypes to Asian targets, *b* = .54, *t*(151) = 4.82, *p* < .001, but not to Black targets, *b* = -.05, *t*(322) = -.76, *p* = .451.

Study 2b: Relative desirability differences for typical Black and Asian targets

- Interaction between target gender and behavior stereotypicality (included)
  - Participant gender did not moderate the two-way interaction between target gender and behavior gender-typicality, *b* < .01, *t*(3755) = .20, *p* = .838, nor did participant race, *b* < .01, *t*(3755) = .06, *p* = .950, or political orientation, b = -.01, t(3736) = 1.16, p = .247. However, the interaction was moderated by age, *b* < .01, *t*(3755) = 3.37, *p* < .001, such that the two-way interaction between target gender and behavior typicality was stronger for older, than for younger, participants.
- Interaction between target race and behavior stereotypicality (not included)
  - Participant gender did not moderate the two-way interaction between target race and behavior gender-typicality, *b* = .02, *t*(3755) = .97 *p* = .332, nor did participant race, *b* = .02, *t*(3755) = .51, *p* = .610. However, the interaction between target race and behavior stereotypicality was moderated by political orientation, *b* = .04, *t*(3774) = 3.18, *p* = .002, such that the interaction was stronger for more conservative participants than for more liberal participants, and was moderated by age, *b* < -.01, *t*(3775) = 2.08, *p* = .038, such that the interaction was stronger for older participants than younger participants.
- Three-way interaction between target gender, behavior stereotypicality, and target race (not included)
  - Participant gender did not moderate the three-way interaction, *b* < .01, *t*(3755) = .191, *p* = .848; nor did participant race, *b* = .07, *t*(3755) = 1.50, *p* = .134, nor political orientation, *b* < -.01, *t*(3736) = .20, *p* = .842. However, the three-way interaction was moderated by participant age, *b* < .01, *t*(3755) = 3.37, *p* < .001, such that the three-way interaction between race, gender, and behavior stereotypicality was stronger for younger participants than for older participants.

Study 3a: Relative expectedness differences for successful working targets

- Participant gender did not moderate the interaction between target gender and behavior typicality, *b* = .03, *t*(3664) = 1.45, *p* = .147; nor did participant race, *b* = -.04, *t*(3664) = .78, *p* = .437. Participant age did moderate this interaction, *b* < -.01, *t*(3645) = 4.20, *p* < .001, such that the interaction was stronger for younger participants than for older participants. Participant political ideology also moderated the interaction, *b* = .07, *t*(3664) = 5.20, *p* < .001, such that the interaction was stronger for more liberal participants than for more conservative participants.

Study 3b: Relative desirability differences for successful working targets (included)

- Participant gender did not moderate the interaction between target gender and behavior typicality, *b* < .01, *t*(3721) = .02, *p* = .981, nor did participant race, *b* = -.03, *t*(3721) = .63, *p* = .531, or age, *b* < -.01, *t*(3702) = .58, *p* = .562. Participant political orientation did moderate the interaction, *b* = -.03, *t*(3702) = -2.41, *p* = .016, such that the interaction was stronger for more liberal participants than for more conservative participants.
